# DVE-1 is a telomere-binding protein and links the NuRD complex to telomere regulation in *C. elegans*

**DOI:** 10.1016/j.isci.2026.117120

**Published:** 2026-08-24

**Authors:** Jan Sluka, Alexandra Blake, Nadezda Podvalnaya, Albert Fradera-Sola, Sabrina Dietz, Lars Teschke, Alejandro Ceron-Noriega, Rosa Herrera-Rodriguez, Valerie Arz, René Ketting, Jan Padeken, Emily Nischwitz, Falk Butter

**Affiliations:** 1 Institute of Molecular Biology, Mainz, Germany; 2 Institute of Molecular Virology and Cell Biology, Friedrich-Loeffler-Institute, Greifswald, Germany; 3 Max Planck Research Group Evolution of Adaptive Immunity in Vertebrates, Max-Planck-Gesellschaft Tuebingen, Tuebingen, Germany; 4 Department of Diagnostic and Interventional Radiology, Heidelberg University Hospital, Heidelberg, Germany

**Keywords:** telomere, *C. elegans*, DVE-1, TERRA, chromatin, NuRD

## Abstract

Telomeres are repetitive DNA sequences at the ends of linear chromosomes bound by specialized proteins. In our previous quantitative proteomics screen for telomere-binding proteins of *Caenorhabditis elegans*, we identified DVE-1, a homolog of mammalian SATB proteins and a transcription factor, as a telomere repeat-binding protein. Here, we validate DVE-1 as a telomere-binding protein in *C. elegans*, demonstrating *in vitro* binding of DVE-1 to the single-stranded C-rich telomeric sequence and *in vivo* co-localization with the telomere-binding protein POT-1. RNA interference-mediated knockdown of *dve-1* resulted in reduced TERRA expression and enhanced compaction of telomeric chromatin. Subsequent transcriptomic and proteomic analyses suggest a role for DVE-1 in the regulation of telomeric chromatin organization. Finally, DVE-1 immunoprecipitation followed by mass spectrometry revealed all the core components of the nucleosome remodeling and deacetylase (NuRD) complex as interaction partners, implicating DVE-1 in the coordination of NuRD complex activity in the context of telomere organization.

## Highlights

• DVE-1 is a confirmed telomere-binding protein
• DVE-1 binds with its C-terminal homeobox to single-stranded C-rich telomeric DNA
• Depletion of DVE-1 reduces TERRA levels and increases telomere chromatin compaction
• IP-MS results implicate DVE-1 as an interactor of the NuRD complex at telomeres

## Graphical abstract

**Figure:**
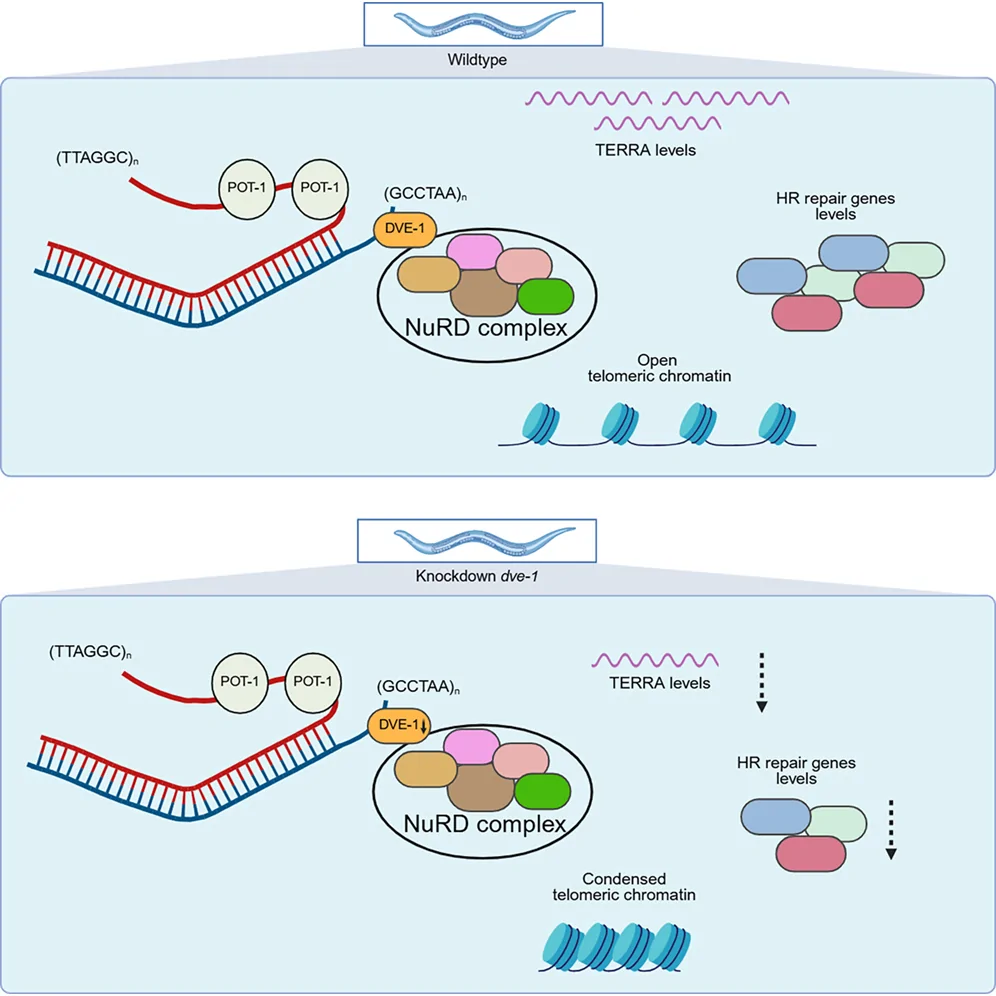

## Teaser

Biological sciences; Nucleic acids; Protein; Molecular biology; Omics; Proteomics; Transcriptomics

## Introduction

Telomeres are present in most eukaryotic species as repetitive DNA sequences at the ends of their linear chromosomes and play crucial roles in maintaining genome stability and ensuring cell viability. Telomeres solve two central problems invoked by chromosome linearity: end replication and end protection.^1^^,^^2^ The end-replication problem arises because cellular DNA polymerases cannot fully replicate the lagging strand at chromosome ends, leading to progressive shortening of telomeres with each cell division.^3^^,^^4^ When telomeres reach a critical minimal length, cells enter replicative senescence.^1^^,^^2^^,^^5^ Telomere maintenance relies on telomerase, a reverse ribonucleoprotein transcriptase responsible for the synthesis of telomeric repeats.^6^ However, certain cancer cells can escape replicative senescence through the activation of the alternative lengthening of telomere (ALT) pathway during cellular immortalization, resulting in highly variable lengths of telomeres.^7^^,^^8^^,^^9^ The end-protection problem occurs because the termini of chromosomes resemble DNA double-strand breaks, which make them susceptible to being mistakenly processed by the DNA damage response machinery.^1^^,^^3^ In most eukaryotic species, this is circumvented by the formation of t-loops, where the single-stranded telomeric overhang folds back and invades the double-stranded telomeric region.^10^^,^^11^

Telomere-binding and telomere-associated proteins are vital to the structural and functional regulation of telomeres.^7^^,^^11^ In mammals, the double-stranded telomere binders TRF1 and TRF2 together with the single-stranded telomere binder POT1 and the additional proteins RAP1, TPP1, and TIN2 form the core shelterin complex.^11^^,^^12^^,^^13^ The proteins CTC1, STN1, and TEN1 are subunits of the single-strand binding CST complex.^14^ Both complexes prevent the inadvertent activation of the DNA damage response and regulate the recruitment of telomerase. Other proteins, such as HMBOX1 and ZBTB48, regulate telomere length, whereas ZNF524 is involved in the telomeric DNA damage response.^15^^,^^16^^,^^17^ The telomere-binding protein ZNF827 recruits the nucleosome remodeling and deacetylase (NuRD) complex to human telomeres, promoting the chromatin remodeling and histone deacetylation, essential for heterochromatin maintenance and ALT in cancer cells.^18^ Interestingly, the binding of NuRD to chromatin is inhibited by active chromatin marks, such as H3K4me2/3, and its binding specificity is further ensured by its interaction with a variety of transcription factors.^19^^,^^20^ The multisubunit NuRD complex consists of six core subunits: HDAC1/2, MTA1/2/3, RBBP4/7, GATAD2A/B, MBD2/3, and CHD3/4.^21^ Together, they can repress transcription, facilitate DNA repair, and regulate differentiation across different cell types.^19^^,^^22^ Dysregulation links NuRD to diseases such as ALT-positive sarcomas, acute myeloid leukemia, and neurodevelopmental disorders.^23^^,^^24^^,^^25^

The shelterin complex and other mammalian telomeric interactors are not evolutionarily conserved, and in different species, other proteins have similar functions.^26^^,^^27^^,^^28^ In *C. elegans*, telomeres consist of (TTAGGC)_n_ repeats that range from 2 to 9 kb in length.^29^^,^^30^ Four OB-folding proteins in *C. elegans* are known, namely, POT-1 (also known as CeOB2), POT-2 (also known as CeOB1), POT-3, and MRT-1, all of which have been shown to bind single-stranded telomeric DNA.^31^^,^^32^^,^^33^^,^^34^ Both C-rich 5′- and G-rich 3′-single-stranded overhangs were reported to be bound by POT-1 or POT-2 and POT-3, respectively.^32^^,^^34^ The loss of POT-1, POT-2, or POT-3 leads to telomere elongation.^32^^,^^34^ In contrast, the absence of MRT-1 leads to progressive telomere shortening and a mortal germline (Mrt) phenotype characterized by sterility in subsequent generations.^31^ TEBP-1 (also known as DTN-1) and TEBP-2 (also known as DTN-2) are two paralogous proteins that bind double-stranded telomeric DNA via a Myb/homeodomain, while their C-terminal tails can be directly bound by the OB-fold of POT-1.^35^^,^^36^ TEBP-1 knockout results in elongated telomeres, whereas TEBP-2 knockout results in telomere shortening and a mortal germline characterized by a progressive loss of fertility across generations.^35^

In our previous quantitative proteomics screen for telomere-binding proteins of *C. elegans*, we identified additional candidates, such as the homeobox protein DVE-1, a homolog of mammalian SATB proteins and a transcription factor that plays a central role in the mitochondrial unfolded protein response.^35^ In this study, we validated DVE-1 as a telomere-binding protein in *C. elegans* that interacts with the NuRD complex, suggesting a link between epigenetic regulation and telomere organization in *C. elegans*.

## Results

### DVE-1 binds to single-stranded telomeric DNA repeats via a homeobox domain

**Figure 1:**
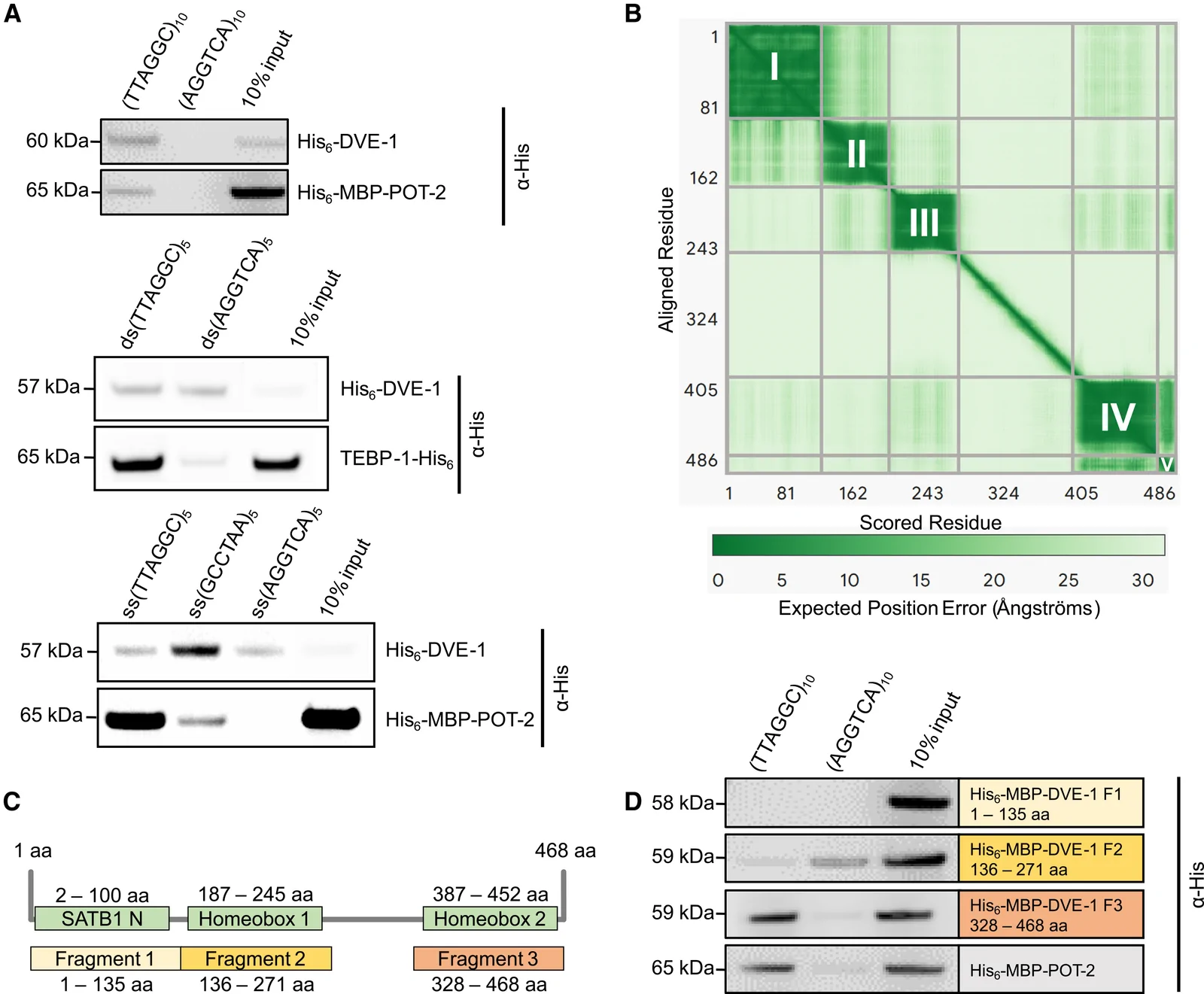
Recombinantly expressed DVE-1 binds telomeric repeats via its homeobox domain (A) Western blots of DNA pulldowns with His_6_-tagged DVE-1 from *E. coli* lysates using concatenated, single-stranded or double-stranded biotinylated DNA oligonucleotide baits with telomeric (TTAGGC) and (GCCTAA) or control (AGGTCA) sequences. Recombinantly expressed POT-2 was used as a known single-strand telomeric binder, and TEBP-1 was used as a known double-strand binder. MBP, maltose-binding protein. The uncropped western blots are shown in Figure S1A of the supplemental information. (B) Predicted aligned error (PAE) plot of AlphaFold3 for DVE-1 and ss (GCCTAA)_3_ DNA. Darker green shading indicates higher model confidence, whereas lighter regions denote areas of increased uncertainty. Four structured regions were predicted, which are indicated in the grid by Roman numerals I–IV and telomeric DNA (V). (C) Schematic of the position of the three generated DVE-1 fragments. Each fragment was fused with an N-terminal His_6_-MBP tag. (D) Western blot of a DNA pulldown experiment as described in (A) with the recombinantly expressed DVE-1 fragments and POT-2 as a positive control. The uncropped western blots are shown in Figure S1B of the supplemental information.

In a quantitative proteomics screen, we previously showed the binding of endogenous DVE-1 to concatenated, biotinylated DNA oligonucleotides with the telomeric (TTAGGC)_n_ sequence.^35^ Here, we carried out DNA pull-down experiments with recombinantly expressed His-tagged DVE-1 and His-MBP-tagged POT-2, a known telomeric binder, followed by western blotting. DVE-1 bound specifically to the telomeric DNA repeats and showed no detectable binding to the (AGGTCA)_10_ control sequence (Figure 1A). To determine the strand specificity of DVE-1, we performed additional DNA pull-down experiments and demonstrated the strong preference of DVE-1 for single-stranded C-rich telomeric DNA (Figure 1A).

AlphaFold3^37^ predicted four structured protein domains, which were annotated with the Conserved Domain Database^38^^,^^39^: an N-terminal region (aa1-100) that matched the SATB1 N-domain with ubiquitin-like structures capable of tetramerization (Figure 1B, box I), a nonannotated structured region (aa101–186) with no similarities to any known domains (Figure 1B, box II), and two homeobox domains (aa187–251 and aa385–458) (Figure 1B, boxes III and IV, respectively). The results of the simulations suggested an interaction between the second homeobox domain (aa385–458) and single-stranded C-rich telomeric DNA repeats (Figure 1B, box V). We recombinantly expressed three different fragments (aa1-aa135, aa136-271, and aa328-468) to experimentally validate the telomere-binding region within DVE-1. Fragment 1 covered the N terminus, and fragment 2 and fragment 3 each covered one of the two predicted homeobox domains (Figure 1C). DNA pull-down experiments followed by western blotting confirmed that only fragment 3, containing homeobox domain 2, preferentially bound to telomeric DNA sequences (Figure 1D).

To determine whether the telomere-binding domain of DVE-1 is conserved beyond *C. elegans*, we performed a multilayered comparative evolutionary analysis integrating orthology inference, gene phylogeny, and domain architecture conservation. This approach enabled discrimination between ancestral conservation and lineage-specific innovation while accounting for the extensive genome rearrangements known to occur within the phylum Nematoda. Across 14 nematode species, DVE-1 is consistently retained as a single-copy gene, exhibiting strict one-to-one orthology across four of the five nematode clades (Figure S4A). The absence of lineage-specific duplications or losses supports the long-term evolutionary stability of this locus. Because one-to-one orthology makes it more plausible that each sequence represents the same evolutionary locus, this pattern supports the conservation of a shared core molecular function.^40^^,^^41^ To further validate vertical inheritance, we compared the DVE-1 gene phylogeny with that of the established nematode species tree. The DVE-1 gene tree closely recapitulates the species-level topology across nematodes (Figure S4A). The concordance between gene and species trees, together with strict one-to-one orthology, is consistent with descent from a single ancestral gene copy and supports the interpretation that the compared sequences correspond to the same gene with a conserved core molecular function.^42^^,^^43^ To assess whether the domain architecture is conserved across nematodes, protein families database (Pfam) and simple modular architecture research tool (SMART) domain coordinates were extracted for each ortholog and merged into a nonredundant, ordered domain architecture. Across representatives of clades I, III, IV, and V, DVE-1 orthologs retained a highly conserved SATB1N-Homeobox1-Homeobox2 architecture (Figure S4B). Conservation of domain number and order among one-to-one orthologs is consistent with structural and functional stability over evolutionary time, whereas changes in domain architecture may indicate functional divergence.^44^ The observed conservation therefore suggests that the telomere-binding domain is not a *C. elegans*-specific feature. Additional bit-score motif representations of the homeobox revealed strong conservation of the amino acids R389 and L390, which are predicted by AlphaFold3 to form hydrogen bonds with the sugar-phosphate backbone and the aromatic base rings (Figures S4C and S4D).

In summary, we demonstrated that DVE-1 directly binds telomeric DNA through its C-terminal homeobox domain, with a preference for single-stranded C-rich telomeric DNA. Its domains and binding motif predicted by AlphaFold3 are highly conserved across different nematode species.

### DVE-1 localizes to telomeres in *C. elegans*

**Figure 2:**
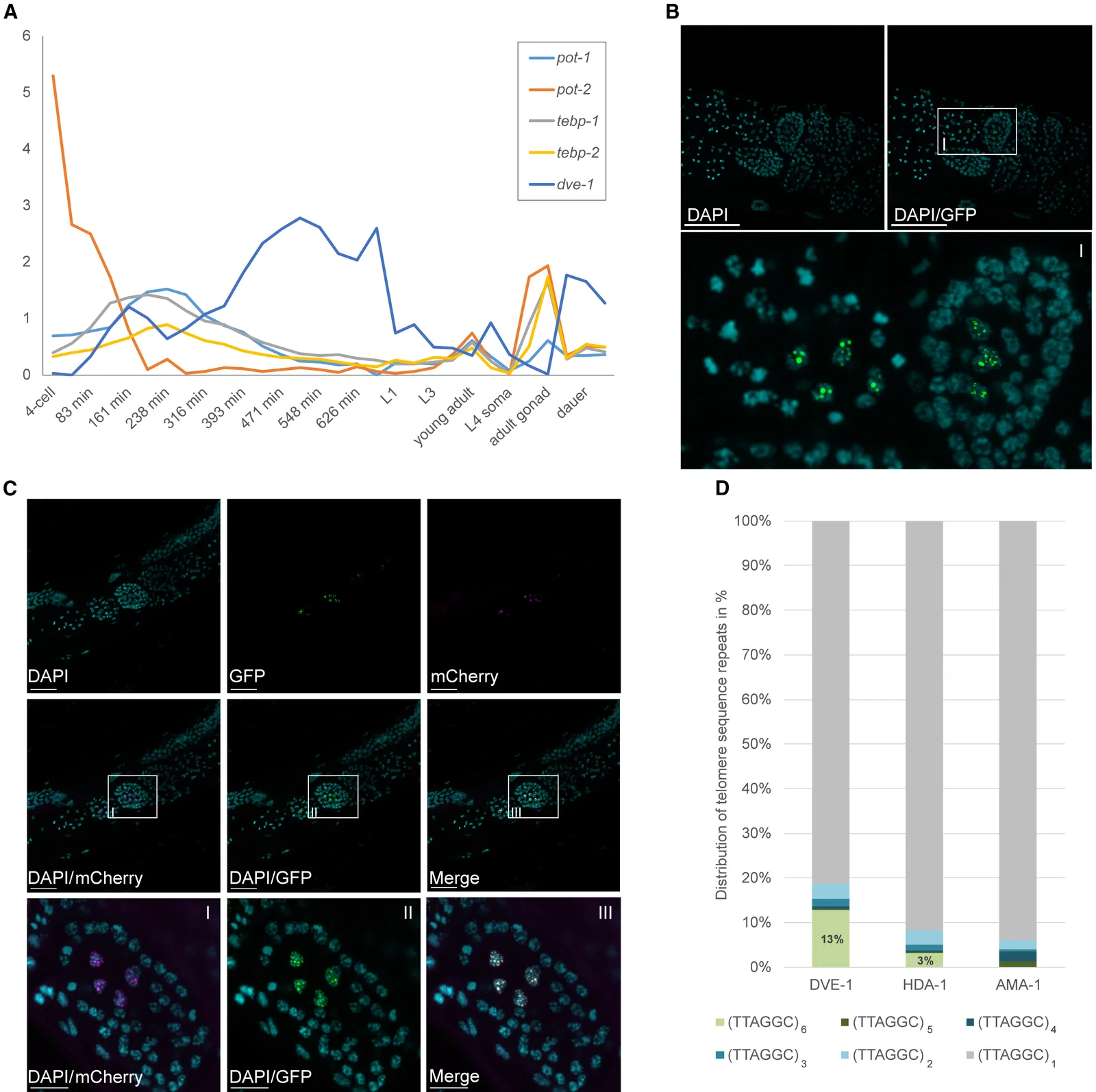
DVE-1 binds *in vivo* to telomeres in late developmental stages of the embryo (A) Gene expression levels in depth of coverage per million reads (dcpm) of DVE-1 and known telomere-binding proteins across developmental stages. Data were sourced from the GExplore database.^45^ (B) Maximum-intensity projections of representative confocal z stacks of embryos in the uterus expressing transgenic DVE-1::GFP in embryonic nuclei. Scale bars, 20 μm (overview). (C) Maximum-intensity projections of representative confocal z stacks of embryos in the germline expressing both transgenic DVE-1::GFP and transgenic POT-1::mCherry. Boxes show nuclear colocalization in embryonic cells. Scale bars, 20 μm (overview) and 5 μm (insets). The inset I shows DNA stained with DAPI and POT-1::mCherry; inset II shows DAPI-stained DNA with DVE-1::GFP; and inset III shows merged DVE-1::GFP, POT-1::mCherry, and DAPI staining. (D) The mean distribution of the detected number of telomere repeats bound to DVE-1, HDA-1, and AMA-1. Data were sourced from the ChIP-seq datasets, GEO: GSE14104^46^ and GEO: GSE258215^47^^,^^48^^,^^49^ (*n* = 2).

As recombinantly expressed DVE-1 preferentially bound to TAAGGC repeat DNA *in vitro* (Figure 1D), we next investigated its expression pattern and telomere association *in vivo*. Gene expression data for DVE-1 and known telomere-binding proteins across developmental stages were obtained from the GExplore database.^45^ DVE-1 exhibited maximal expression during late embryogenesis, a stage in which known telomere-binding proteins such as TEBP-1, TEBP-2, POT-1, and POT-2 were expressed at relatively low levels (Figure 2A). This expression pattern is consistent with the confocal microscopy images obtained for the transgenic DVE-1::GFP strain, which revealed distinct nuclear foci in late-stage embryos (Figure 2B). To test whether these foci corresponded to telomeres, we crossed DVE-1::GFP and POT-1::mCherry single-copy transgenic strains.^32^ In heterozygous embryos, 95% of DVE-1::GFP foci colocalized with POT-1::mCherry (Figure 2C; Figure S3), demonstrating the localization of DVE-1 to telomeres *in vivo*.

To further confirm telomeric binding, we reexamined previously published ChIP-seq datasets to quantify the enrichment of telomeric repeats by DVE-1 and the histone deacetylase HDA-1 as a negative control.^46^ Across an average of 15,784,641 total reads, DVE-1 immunoprecipitation contained 89,606 reads with six or more consecutive telomeric repeats (TTAGGC). This corresponds to a fourfold enrichment of telomeric repeats bound by DVE-1 compared with HDA-1, which had an average of 21,328 such reads from 17,136,564 in total (Figure 2D). For further control, the DNA-directed RNA polymerase II subunit AMA-1 (also known as rpb-1) does not contain telomeric repeat sequences within its read profile (Figure 2D).^47^^,^^48^^,^^49^ Together, these data demonstrated that DVE-1 is strongly expressed during late embryogenesis, is localized to telomeres *in vivo* and purified a substantial proportion of telomere-repeat-containing DNA.

### DVE-1 depletion compacts telomeric chromatin and disrupts TERRA and DNA damage response gene expression

**Figure 3:**
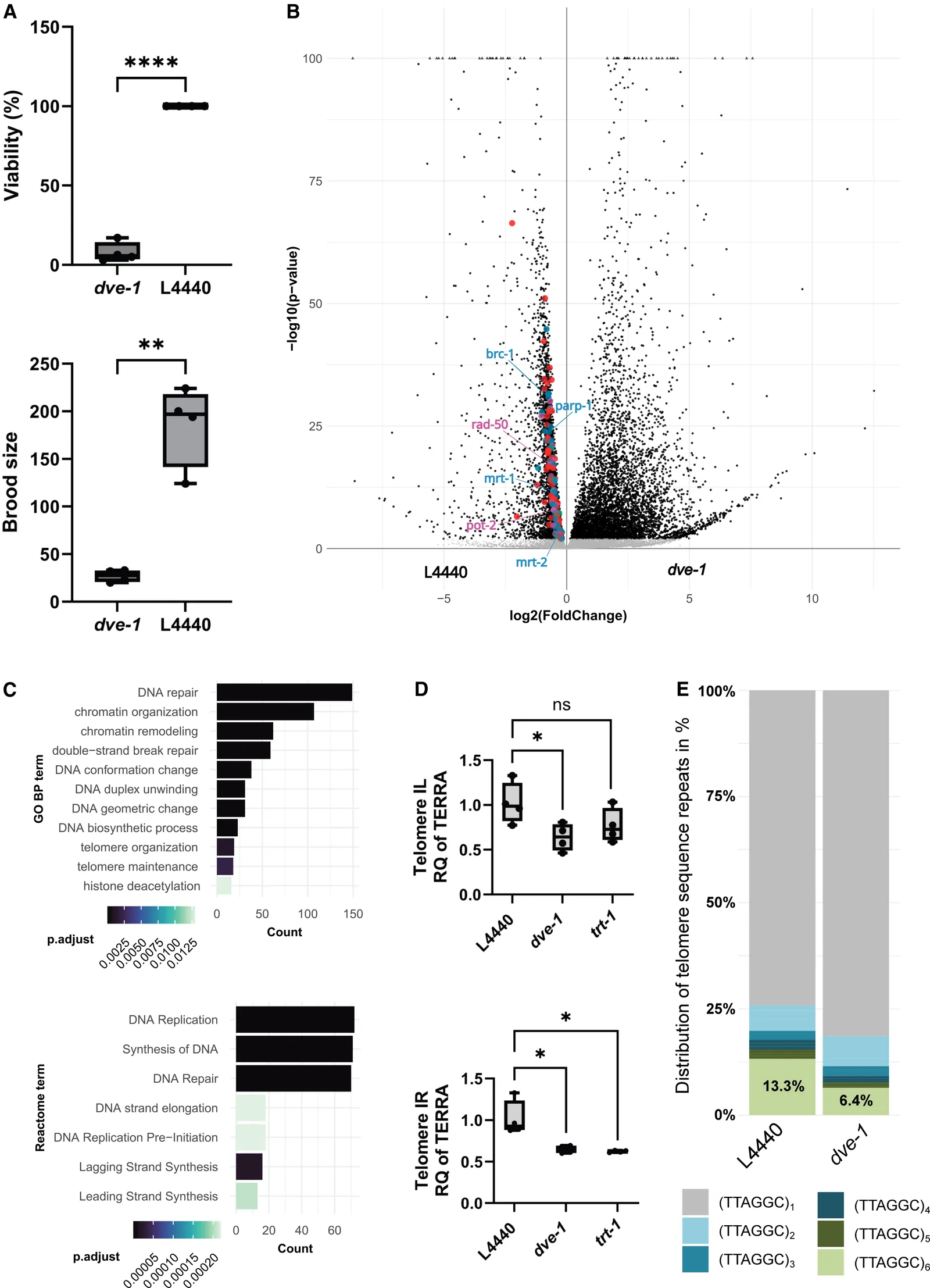
DVE-1 is essential for embryonic viability and brood size and influences TERRA expression, telomeric chromatin accessibility, and the expression of genes involved in DNA repair, chromatin, and telomere organization (A) Quantification of the viability of the F1 progeny at the L1 stage and the brood size of the F0 generation. The results are shown as a boxplot with whiskers from minimum to maximum. *p* values were calculated by two-sided Welch’s *t* test (∗∗*p* < 0.005; ∗∗∗∗*p* < 0.0001) (*n* = 4). (B) Volcano plot of poly(A)-enriched RNA sequencing data from synchronized gravid adult worms with *dve-1* knockdown versus the L4440 empty vector negative control (*n* = 4). Differentially expressed genes (DEGs) whose adjusted *p* value (false discovery rate) was <0.01 were selected and are highlighted as black dots. Background genes are depicted in gray. Enriched genes from the following GO BP terms are colored: “DNA repair” (blue), “chromatin organization” (red), “telomere organization” (green), and the genes shared between the terms are highlighted in pink. (C) GO BP and Reactome analyses of genes whose expression is downregulated upon *dve-1* knockdown, based on the Gene Ontology and Reactome databases.^52^^,^^53^ Terms found among the enriched genes were tested for overrepresentation with an adjusted *p* value (false discovery rate) < 0.05 (Fisher’s exact test). (D) RT-qPCR analysis of TERRA expression on telomere IL and IR in wild-type gravid adult worms with *dve-1* knockdown versus the L4440 empty vector negative control and the *trt-1* mutant strain. The results are shown as boxplots with whiskers from minimum to maximum. *p* values were calculated by two-tailed Welch’s *t* test (∗*p* < 0.05) (*n* = 4). (E) The mean distribution of the number of detected telomere repeats according to ATAC-seq of synchronized gravid adult worms with *dve-1* knockdown versus the L4440 empty vector negative control (*n* = 3).

To assess the potential functions of DVE-1 in *C. elegans*, we examined the effects of *dve-1* knockdown, as the knockout is embryonic lethal.^50^ Stage-synchronized wild-type worms were fed from the L1 stage on with *E. coli* expressing double-stranded RNA targeting *dve-1*.^51^ Strong DVE-1::GFP fluorescence revealed robust DVE-1::GFP expression throughout all the embryos in the L4440 empty vector negative control group, whereas the *dve-1* RNAi-treated group showed a nearly complete absence of DVE-1::GFP signal in the embryos, confirming efficient knockdown at the protein level (Figure S1A). Next, we investigated the phenotypic effects of *dve-1* knockdown. Quantification of F1 progeny viability demonstrated that *dve-1* knockdown resulted in a statistically significant reduction in the number of viable L1 larvae (*p* < 0.0001, Welch’s *t* test, two-tailed). Brood size was also significantly decreased under knockdown conditions compared to the negative control (*p* = 0.0046, Welch’s *t* test, two-tailed). In contrast, the negative control resulted in no mortality with the expected brood size, with all the progeny surviving to the L1 stage and beyond (Figure 3A). Whether a loss of telomere stability or other effects of DVE-1 deficiency cause these phenotypes is unknown.

To further characterize the effects of *dve-1* knockdown, we investigated transcriptional and translational changes in the worms. Total RNA and protein were collected from stage-synchronized hermaphroditic gravid adults. In the results of the transcriptomic analysis of *dve-1* knockdown versus the L4440 empty vector negative control, 10,002 transcribed genes were identified, 4,735 of which were significantly downregulated (adjusted *p* value <0.01) (Figure 3B). These downregulated genes were tested for overrepresentation of biological process gene ontology terms. The results revealed a reduction in various terms, including DNA repair, chromatin organization, chromatin remodeling, and telomere organization and maintenance. Additionally, a similar functional analysis for the Reactome pathway terms revealed an overrepresentation of the DNA replication and DNA strand elongation pathways (Figure 3D). Among the 149 downregulated genes involved in DNA repair, homologous recombination (HR) repair genes, such as *brc-1*, *parp-1*, and *rad-50*, were found. Furthermore, the expression of 19 telomere organization genes, including *mrt-1*, *mrt-2* and *pot-2*, was also reduced (Figure 3C). In addition to the transcriptome, we measured the proteome of worms from the same population. In total, 2,922 proteins were identified, of which 519 were more than 2-fold downregulated and 65 were more than 2-fold upregulated (*p* value <0.05) (Figure S1B). Overlapping reduced expression was observed in both the transcriptome and proteome for 1,467 genes (*p* value <0.01) (Figure S1C). The Argonaut genes, *wago-1* and *hrde-1*, which are involved in subtelomere and telomere organization, were included in this overlap.^54^

To investigate the effects of DVE-1 depletion on telomere maintenance processes, we measured the expression levels of the long non-coding RNA TERRA using quantitative RT-PCR (RT-qPCR). The *trt-1* strain served as a positive control and is known to reduce TERRA expression.^55^ TERRA levels were significantly reduced at telomere IR and IL upon the knockdown of *dve-1* (Figure 3D). Additional assays for transposase-accessible chromatin using sequencing (ATAC-seq) revealed that the number of long telomeric repeats was reduced by more than half under *dve-1* knockdown conditions (Figure 3E), indicating that the accessibility of telomeric chromatin decreases significantly when DVE-1 levels are reduced. The reduced expression of TERRA and accessibility of telomeric chromatin are consistent with our observations of chromatin organization changes in the transcriptome and link DVE-1 to a regulatory role of chromatin remodeling at telomeres.

Collectively, these results show that DVE-1 strongly affects chromatin modifiers, DNA damage response factors, and proteins involved in telomere organization. Its depletion has a significant effect on the expression levels of TERRA and the accessibility of chromatin at telomeres, suggesting that it plays a regulatory role in telomeric chromatin organization. However, we cannot distinguish whether these transcriptional and phenotypical effects are direct or secondary consequences of DVE-1 deficiency or whether they reflect specific telomeric effects.

### DVE-1 is an interactor of the NuRD complex in *C. elegans*

**Figure 4:**
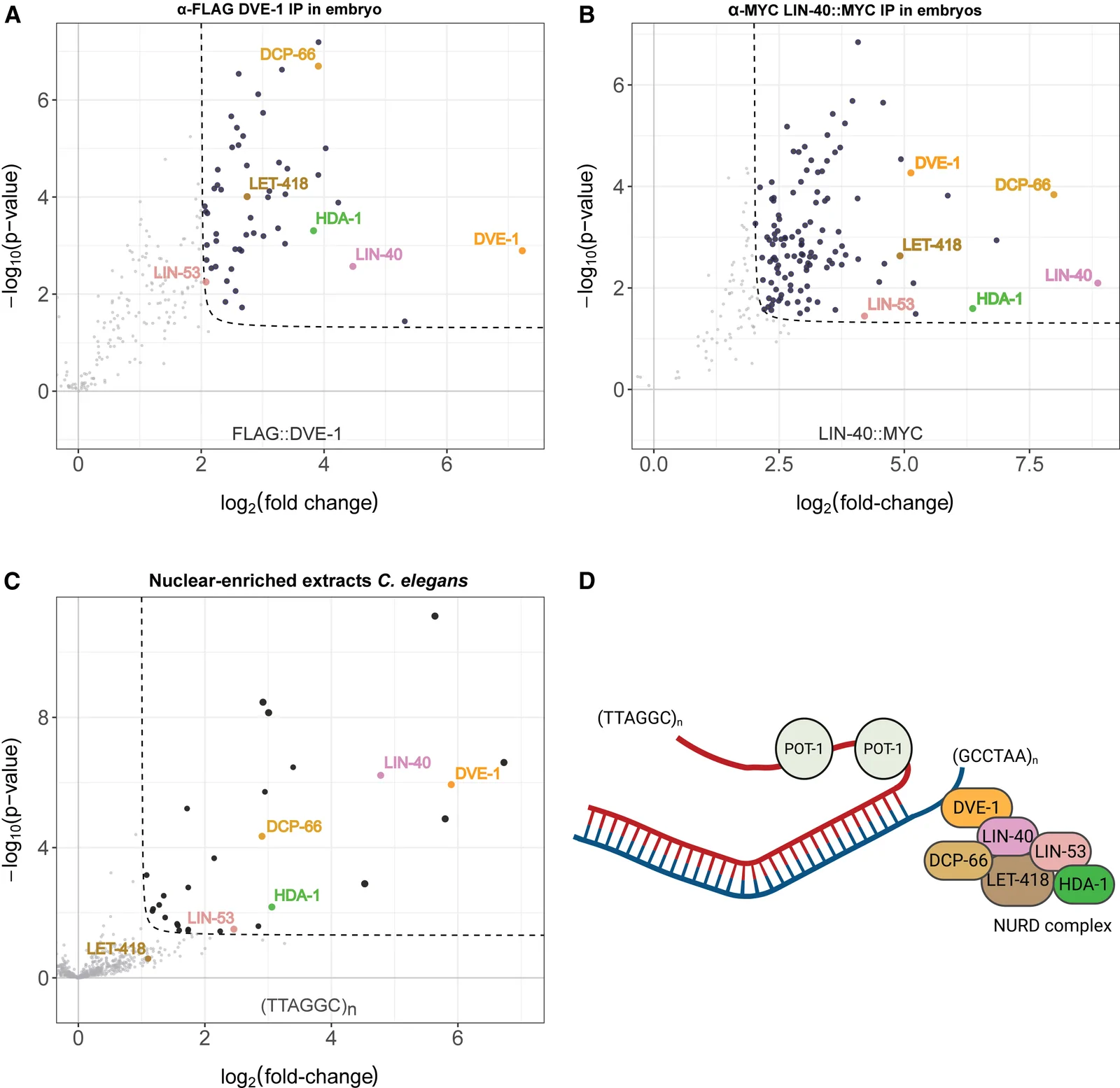
DVE-1 immunoprecipitates with NuRD complex members (A and B) Volcano plots showing the results of the quantitative proteomic analysis of (A) FLAG::DVE-1 and (B) LIN-40::MYC immunoprecipitates with embryo lysates. IPs were performed in quadruplicates. The log_2_-fold enrichment of proteins that interact with the bait protein versus the control is presented on the *x* axis. The *y* axis shows the −log_10_*p* values (Welch *t* test) of enrichment across replicates. More than 4-fold enriched proteins with *p* values < 0.05 are presented as black dots, the background proteins are shown as gray dots, and the NuRD complex is highlighted. (C) Volcano plot representing label-free proteomic quantification of pulldowns with biotinylated, concatenated oligonucleotide baits of telomeric DNA sequence (TTAGGC)_n_ or control DNA sequence (AGGTCA)_n_. Pulldowns were performed with nuclear extracts from synchronized gravid adult animals in octuplicates per condition (two biological replicates, each with four technical replicates) by Dietz et al.^35^ Enriched proteins (threshold: 2-fold, *p* < 0.05) are shown as black dots and enriched proteins of interest are highlighted. Background proteins are represented as gray dots. (D) Proposed working model: DVE-1 binds to single-stranded C-rich telomeric DNA. The NuRD complex subunits LIN-40, HDA-1, LIN-53, DCP-66, and LET-418 are associated with DVE-1. Created in BioRender. Sluka, J. (2026) https://BioRender.com/29w24zf.

To identify the interaction partners of DVE-1, we endogenously tagged DVE-1 at its N terminus with a FLAG tag by CRISPR-Cas9 genome editing and confirmed its expression by western blotting (Figure S2A). We then performed immunoprecipitation followed by quantitative mass spectrometry (IP-MS) with the FLAG::DVE-1 embryonic protein extract (Figure 4A). A total of 264 proteins were identified, 53 of which were positively enriched. Among these proteins, DVE-1 was highly enriched, together with all the core members of the NuRD complex^56^: LIN-40, LET-418, HDA-1, DCP-66, and LIN-53. No known telomere-binding proteins were identified. To independently confirm the immunoprecipitation of DVE-1 with the NuRD complex, we generated an endogenously tagged LIN-40::MYC strain by CRISPR-Cas9 genome editing and confirmed its expression by western blotting (Figure S2B). We subsequently performed IP-MS with the embryo protein extract (Figure 4B). In total, 207 proteins were identified, 119 of which were positively enriched. LIN-40 was highly enriched and co-immunoprecipitated with all known NuRD complex members as well as with DVE-1.

To confirm which NuRD complex members are enriched at telomeric repeats, we reanalyzed our original telomere screen with the WormBase protein database version WS269 and 2-fold enriched proteins with an adjusted *p* value<  0.05.^35^^,^^57^ We found 805 proteins, 29 of which were statistically significantly enriched for telomeric repeats (Figure 4C). All six core members of the NuRD complex detected by FLAG::DVE-1 IP-MS were likewise identified and enriched in a previously published telomeric DNA pulldown.^35^ These findings establish a direct link between the NuRD complex and telomere biology in *C. elegans*.

## Discussion

Here, we demonstrated that DVE-1 is an essential telomere-binding protein in *C. elegans*. We provide evidence that DVE-1 directly binds telomeric DNA via its C-terminal homeobox domain. In addition, our *in vivo* confocal microscopy images demonstrate the telomeric localization of DVE-1 by co-localization with POT-1. The results of the ChIP-seq data support these findings because of the enrichment of a large ratio of telomeric repeat sequences bound to DVE-1. Depletion of DVE-1 decreased TERRA expression and led to regulatory changes in DNA repair and chromatin regulation genes. Finally, IP-MS experiments confirmed that DVE-1 interacts with the NuRD complex.

Previous research has shown that the accumulation of DVE-1∷GFP in subnuclear foci is dependent on the heterochromatin proteins SETDB1 homolog (MET-2) and ATF7IP homolog (LIN-65), at least in the context of mitochondrial stress. Whether this is also the case under nonstress conditions is not clear, but it highlights a potential connection between DVE-1 and condensed heterochromatin.^58^ Regarding the role of DVE-1 in mitochondrial stress responses, a connection of DVE-1 to the NuRD complex members LIN-40, LIN-53, LET-418, HDA-1, and DCP-66 has also been reported.^46^^,^^56^ The NuRD complex is an evolutionarily conserved, multisubunit chromatin remodeler found in diverse eukaryotes.^59^ In *C. elegans*, the NuRD complex consists of the core subunits HDA-1 (histone deacetylase/HDAC homolog), LIN-53 (histone chaperone/RBBP homolog), LIN-40 (MTA homolog), and the Mi2 ATPase homologs CHD-3 and LET-418.^19^^,^^56^^,^^60^^,^^61^^,^^62^ The composition varies in a developmental and tissue-specific manner, with LET-418 and CHD-3 forming separate NuRD variants.^61^^,^^63^ Functionally, the NuRD complex regulates gene repression, developmental patterning, chromatin remodeling, and metabolic stress responses, with mutations in its subunits affecting both lifespan and stress responses.^19^^,^^56^^,^^63^^,^^64^ In our previous quantitative proteomics screen, we originally detected enrichment of DVE-1 and LIN-40 on telomeric DNA.^35^ Reanalysis of these data also retrospectively revealed enrichment of LIN-40, LIN-53, LET-418, HDA-1 and DCP-66. The detection of these NuRD complex members by DVE-1 IP-MS, together with the reciprocal co-precipitation of DVE-1 by LIN-40, implicates DVE-1 as an interactor of the NuRD complex. Thus, we identified a link between the NuRD complex and telomeres in *C. elegans*, in which DVE-1 connects the core NuRD complex members LIN-40, LIN-53, LET-418, HDA-1, and DCP-66 to telomeric DNA.

The role of the NuRD complex at telomeres has already been well documented in human cells.^18^ In ALT positive cells, the NuRD complex is recruited by ZNF827 to telomeres, where it remodels the surrounding chromatin and changes its protein composition.^18^ The histone deacetylase (HDAC) activity of NuRD-ZNF827 at ALT telomeres causes histone hypoacetylation, while the binding of shelterin proteins to telomeres decreases. Telomere-telomere interactions are enhanced, and HR proteins, such as BRIT1 or BRC1, are recruited.^18^^,^^65^ A recent study has shown that the NuRD complex is also required in *C. elegans* for effective HR at DNA double-strand breaks.^66^ This finding aligns with the transcriptome and proteome results of the *dve-1* knockdown, in which we detected a reduction of the *brca1* ortholog *brc-1* and other HR-repair genes, such as *parp-1* and *rad-50*. Our observation of the reduction in TERRA expression levels further suggests that DVE-1 plays a central role in telomere dynamics in *C. elegans*. TERRA has already been shown to serve as a marker of ALT activity, as its expression is elevated both before and during ALT.^55^ Together with the recruitment of the NuRD complex to telomeres, we propose that DVE-1 functions analogously to ZNF827 in humans and remodels telomeric chromatin along with the NuRD complex. DVE-1, in association with the NuRD complex, might reduce the binding of the two TEBP proteins as well as POT-2 and recruit HR proteins to enable ALT initiation. Consistent with this model, DVE-1 depletion is expected to reduce the presence of NuRD at telomeres, resulting in decreased chromatin accessibility, as observed by our ATAC-seq data, and consequently impaired ALT activity, potentially promoting a negative feedback loop to TERRA expression. Whether the reduction in TERRA levels represents an indirect effect of a negative feedback loop or a direct effect of DVE-1 remains unclear and requires further investigation. The underlying mechanism, however, appears to be evolutionarily preserved outside of human cells.

Further investigations of the role of DVE-1 in the chromatin remodeling of telomeres are needed. Our study implicates DVE-1 as the bridge between the NuRD complex and telomeres, but further elucidation of the mechanistic role of the NuRD complex at telomeres is needed.

### Limitations of the study

ChIP-seq analysis of HDA-1 revealed a small fraction of long telomeric repeats. Although this pattern is suboptimal for use as a negative control, it is consistent with the predicted association of the NuRD complex with telomeric regions. Such proximity may result in the co-purification of telomeric DNA fragments during the ChIP-seq procedure, despite the absence of evidence for direct telomere binding by HDA-1. In contrast, our second negative control, AMA-1, which is unrelated to the NuRD complex, showed no enrichment for long telomeric repeats. Ideally, these control datasets would have originated from the same experimental batch as the DVE-1 and HDA-1 ChIP assays. Notably, even under these conditions, the DVE-1 ChIP-seq data revealed approximately fourfold more long telomeric repeats than HDA-1 did, providing strong support for direct *in vivo* binding of DVE-1 to telomeres. The increased condensation of chromatin regions containing long telomeric repeat sequences observed in the ATAC-seq analysis does not allow for a distinction between telomere-proximal and interstitial repeat sequences. However, the reduced TERRA expression levels suggest a change in chromatin structure within the telomeric regions.

## Resource availability

### Lead contact

Requests for further information and resources should be directed to and will be fulfilled by the lead contact, Falk Butter (falk.butter@fli.de).

### Materials availability

Reagents generated in this study will be made available on request, but we may require a payment and/or a completed materials transfer agreement if there is potential for commercial application.

### Data and code availability

• All standardized datasets are deposited and publicly available. The mass spectrometry proteomics data are available at the ProteomeXchange Consortium under the accession number PRIDE: PXD069391. RNA sequencing data generated in this study have been deposited to SRA: SRP636574. ATAC-seq data are accessible with GEO: GSE333583. This paper analyzes existing, publicly available ChIP-seq data: GEO: GSE14104^46^ and GEO: GSE258215.^47^^,^^48^^,^^49^
• This paper does not report original code.
• All other data reported in the manuscript will be shared by the lead contact upon request. Any additional information required to reanalyze the data reported in this paper is available from the lead contact upon request

## Acknowledgments

We thank all members of the Butter Lab for insightful discussions and valuable comments on this work. We are especially grateful to Rachel Mullner for her excellent laboratory management. We also thank the IMB Protein Production Core Facility, especially Martin Möckel and Sabine Heinen, for the supply of Tn5 transposase and their expert advice on the expression of recombinant proteins. We further acknowledge the IMB Media Lab for the media and reagents provided. We thank the IMB Flow Cytometry Core Facility, especially Stefanie Möckel and Stephanie Nick, for their assistance with the experimental setup and analysis. In addition, we thank the Genomics Core Facility for their support and wish to express our appreciation and the use of their instruments. The study received funding from the 10.13039/501100001659German Research Foundation (project no. 329045328) supported by the Illumina NextSeq500. We thank the Ahmed laboratory for providing strains. Some strains were provided by the CGC, which is funded by the NIH 10.13039/100016958Office of Research Infrastructure Programs (P40 OD010440). The graphical abstract was created in BioRender. Sluka, J. (2026) https://BioRender.com/hvoimeu. J.P. and V.A. were funded by the 10.13039/501100001659Deutsche Forschungsgemeinschaft (10.13039/501100001659DFG, 10.13039/501100001659German Research Foundation; project ID 393547839 – SFB 1361). J.P. and R.H.-R. were also funded by the 10.13039/501100001659Deutsche Forschungsgemeinschaft (10.13039/501100001659DFG, 10.13039/501100001659German Research Foundation; project ID 491145305 – GRK 2859). This project was funded by the 10.13039/501100001659Deutsche Forschungsgemeinschaft (DFG, 10.13039/501100001659German Research Foundation; project ID BU 2996/17-1).

## Author contributions

Conceptualization, F.B.; investigation, J.S., A.B., N.P., S.D., L.T., E.N., R.H.-R., and V.A.; formal analysis, J.S., A.F.-S., A.C.-N., and J.P.; visualization, J.S., A.F.-S., A.C.-N., and J.P.; writing – original draft, J.S., F.B., and E.N.; writing – review and editing, all authors contributed; supervision, E.N. and F.B.; project administration, F.B.; funding acquisition, F.B.

## Declaration of interests

The authors declare no competing interests.

## Declaration of generative AI and AI-assisted technologies in the writing process

During the preparation of this work, the authors used perplexity.ai and ChatGPT (GPT-4) to optimize readability. After using this tool or service, the authors reviewed and edited the content as needed and take full responsibility for the content of the publication.

## STAR★Methods

### Key resources table

**Table:** 

REAGENT or RESOURCE	SOURCE	IDENTIFIER
**Antibodies**		

anti-His-HRP-conjugated antibody	Qiagen	Cat#34460; RRID: AB_3699318
FLAG antibody mouse M2	Sigma-Aldrich	Cat#F1804; RRID: AB_262044
MYC antibody clone 9E10	IMB Mainz Protein CF	N/A

**Bacterial and virus strains**		

BL21(DE3) arctic express cells	Agilent	Cat#230192
HT115(DE3)	Caenorhabditis Genetics Center (CGC)	https://cgc.umn.edu/strain/HT115(DE3)

**Chemicals, peptides, and recombinant proteins**		

cOmplete™ EDTA-free Protease Inhibitor Cocktail	Roche	Cat#4693132001
Dynabeads™ MyOne™ Streptavidin C1	Thermo Scientific	Cat#65002
Dynabeads™ Protein G	Invitrogen	Cat#10004D
DNase I	Zymo Research	Cat# E1010
SuperScript IV Reverse Transcriptase	Invitrogen	Cat#18090010
Trypsin, MS approved, from porcine pancreas	Serva	Cat#37286

**Critical commercial assays**		

Power SYBR Green PCR Master Mix	Invitrogen	Cat#4368706
Ni-NTA Spin Kit	Qiagen	Cat#31314
Illumina TruSeq stranded mRNA LP Sample Prep Kit	Illumina	Cat#20020594

**Deposited data**		

ChIP-Seq data	Shao et al.^46^	GEO: GSE14104
ChIP-Seq data	ENCODE Project^47^^,^^48^^,^^49^	GEO: GSE258215
RNA-Seq data	This paper	SRA: SRP636574
Proteomics data	This paper	PRIDE: PXD069391
ATAC-Seq data	This paper	GEO: GSE333583

**Experimental models: Organisms/strains**		

RFK1848 dve-1(xf398[3xflag:dve-1]), X	This paper	N/A
RFK1847 lin-40(xf397[lin-40:3myc]), V	This paper	N/A
N2 (Bristol)	CGC	https://cgc.umn.edu/strain/N2%20(ancestral)
SJ4197 [dve-1p:dve-1:GFP]	CGC	https://cgc.umn.edu/strain/SJ4197
YA1197 ypIn2 (Pdaz-1:pot-1:mCherry:tbb-2utr)	Shtessel et al.^32^	N/A

**Oligonucleotides**		

See Table S1 for all oligonucleotides utilized for cloning, sequencing, DNA pulldowns and other assays	This paper	N/A

**Recombinant DNA**		

L4440 empty vector	N/A	https://www.addgene.org/1654/

**Software and algorithms**		

CRISPOR platform	Concordet et al.^67^	http://crispor.tefor.net
STAR aligner version 2.7.3a	Dobin et al.^68^	https://github.com/alexdobin/STAR/releases
featureCounts version 1.6.2	Liao et al.^69^	http://subread.sourceforge.net
MultiQC version 1.7	Ewels et al.^70^	http://multiqc.info
MaxQuant	Max-Planck Institute of Biochemistry, Computational Systems Biochemistry	https://maxquant.org/
R	The R Foundation	https://www.r-project.org/

**Other**		

2100 Bioanalyzer	Agilent	P#G2939BA
Illumina NextSeq 2000	Illumina	N/A
Nanocapillary column 25 cm length, 75 μm inner diameter	New Objective	N/A
Aurora Ultimate CSI 25 × 75 C18 UHPLC column	Ionopticks	Cat#AUR4-25075C18-CSI
EASY-nLC 1000	Thermo Scientific	P#LC120
Q Exactive Plus mass spectrometer	Thermo Scientific	Cat#IQLAAEGAAPFALGMBDK
TimsTOF HT	Bruker	https://www.bruker.com/en/products-and-solutions/mass-spectrometry/timstof/timstof-ht.html
NanoElute 2 HPLC system	Bruker	N/A
TCS SP5 Leica confocal microscope	Leica	https://www.leica-microsystems.com/products/confocal-microscopes/p/leica-tcs-sp5/
Leica STELLARIS 8 FALCON	Leica	https://www.leica-microsystems.com/products/confocal-microscopes/p/stellaris-8-falcon/?country=DE

### Experimental model and study participant details

#### C. elegans

Embryo extraction was performed with gravid adults. Worms were synchronized and grown on egg plates until they reached the gravid adult stage and laid eggs. Then, worms were washed with M9 buffer 3 times, bleached in bleach solution (0.8% Hypochloride Solution (Sigma-Aldrich), 0.3 M NaOH) until only embryos and no whole worms were visible, washed again in M9 buffer and flash-frozen in lysis buffer (25 mM Tris (pH 7.5), 150 mM NaCl, 3 mM MgCl_2_, 1 mM DTT, 2% Triton *X*-100, and 1*x* complete protease inhibitors, Roche) with liquid nitrogen into balls. Worm balls were ground into a powder with pestle and mortar. The powder was transferred into a precooled glass douncer (Kimble), and sheared over 30 strokes with piston B. The lysate was centrifuged at maximum speed for 15 min at 4°C. The supernatant was flash-frozen in liquid nitrogen and stored at −80°C. For proteome measurement, 20 synchronized gravid adult worms per replicate were directly lysed in 1*x* LDS and proceeded with in-gel digest.

#### Bacteria

*E. coli* strain HT115(DE3) pre-cultures for RNAi were grown at 37°C and 180 rpm in LB-media containing tetracycline (10 μg/mL) and ampicillin (100 μg/mL). The *E. coli* strain BL21(DE3) arctic express pre-cultures were grown in LB-media at 37°C and 180 rpm and the expression cultures at 12°C and 180 rpm in LB-media supplemented with 1 mM IPTG.

### Method details

#### Oligonucleotides

All the oligonucleotides utilized for cloning, sequencing, DNA pulldowns and other assays are listed in Table S1 Oligonucleotides.

#### Telomere pulldown experiments

Telomere pulldown experiments were carried out as described by Dietz et al. (2021).^35^ In summary, biotinylated oligonucleotides (Metabion) were used, each containing either the G-rich telomeric sequence, the C-rich telomeric sequence, or a control sequence. Double-stranded oligonucleotides were generated by mixing the biotinylated forward oligonucleotide in a 1:1 ratio with the corresponding non-biotinylated reverse complement, followed by the addition of annealing buffer (200 mM Tris-HCl (pH 8.0), 100 mM MgCl_2_, and 1 M KCl). This mixture was incubated at 80°C for 5 min and then allowed to cool gradually to room temperature. Single-stranded oligonucleotides were prepared in the same manner, substituting the reverse complement with ddH_2_O. The biotinylated DNA and Dynabeads MyOne Streptavidin C1 (Thermo Scientific, #65002) were combined with protein binding buffer (PBB) (50 mM Tris/HCl (pH 7.5), 150 mM NaCl, 0.5% NP-40, 5 mM MgCl_2_, and 1 mM DTT) and incubated at room temperature for 30 min on a rotating wheel to immobilize the DNA onto the beads. After three washes with PBB buffer, the DNA-coupled beads were resuspended in PBB buffer, and Salmon sperm DNA (10 mg/mL, Ambion, #AM9680) was added at a 1:1000 dilution as a competitor to prevent nonspecific DNA binding. Pulldowns were performed using the respective amounts of protein extract: 500 μg of *C. elegans* embryo extract for mass spectrometry and 5–20 μg of *E. coli* extract for western blot analyses. The samples were then incubated at 4°C on a rotating wheel for 90 min. Following incubation, the beads were washed three times with PBB buffer and resuspended in 1× loading buffer (4× NuPAGE LDS sample buffer; Thermo Scientific; #NP0008) supplemented with 100 mM DTT. For elution, the samples were boiled at 70°C for 10 min, loaded onto a gel and processed for mass spectrometry or western blot analysis. For the pulldown-MS experiments, samples were prepared in technical quadruplicates (LFQ) per condition.

#### Immunoprecipitation experiments

Immunoprecipitation (IP) experiments were carried out using 30 μL Dynabeads Protein G (Invitrogen, #10004D) per IP sample. After three washes with washing buffer (25 mM Tris/HCl (pH 7.5), 150 mM NaCl, 51.5 mM MgCl_2_, and 1 mM DTT), the beads were resuspended in fresh washing buffer, and added to 500 μg of the respective *C. elegans* embryo extract supplemented with either FLAG antibody mouse M2 at a 1:1000 dilution (Sigma-Aldrich, #F1804) or MYC antibody clone 9E10 at a 1:1000 dilution (IMB Mainz Protein CF). The samples were incubated at 4°C on a rotating wheel for 3 h. Following incubation, the beads were washed three times with washing buffer and resuspended in 1× loading buffer (4× NuPAGE LDS sample buffer; Thermo Scientific; #NP0008) supplemented with 100 mM DTT. For elution, the samples were boiled at 70°C for 10 min.

#### Expression and purification of recombinant protein from *E. coli*

Cloning was performed as described by Dietz et al. (2021).^35^ Pre-cultures of BL21(DE3) arctic express cells (Agilent, #230192) in 6 mL of LB medium were inoculated and shaken overnight at 37°C and 180 rpm with the respective selection antibiotics of the expression plasmid. After overnight incubation, the pre-culture was diluted to a final volume of 100 mL in LB media that included the respective selection antibiotics. Cells were grown at 37°C and 180 rpm until the OD600 was 0.6 and then cooled on ice, and recombinant expression was induced by adding IPTG to a final concentration of 1 mM. The expression culture was incubated overnight at 12°C and 180 rpm. The cells were harvested the next day at 4000 × g for 10 min at 4°C. The resulting pellet was resuspended in 6 mL of lysis buffer (300 mM NaCl, 25 mM Tris-HCl (pH 7.5), 50 mM imidazole) supplemented with 1 mM DTT and protease inhibitor cocktail tablets (Roche, #4693132001)). Cells were lysed three times on ice by sonication with a Branson Sonifier for 3 min, followed by a 3-min pause on ice (duty cycle: 50%, output control 25%). The lysate was then transferred and centrifuged in 2 mL reaction tubes at 21,000 × g for 10 min at 4°C to separate the soluble parts from the insoluble parts. His-tagged proteins were purified from the soluble lysate using a Ni-NTA Spin Kit (Qiagen, #31314) according to the manufacturer’s instructions.

#### AlphaFold predictions

Structural predictions were conducted using AlphaFold3^37^ to model the DVE-1 protein (UniProt ID: Q86MI0) in complex with single-stranded telomeric DNA consisting of three GCCTAA repeats and one magnesium ion. Model confidence was assessed using the predicted inter-chain TM-score (ipTM = 0.55) and predicted TM-score (pTM = 0.28), as provided by the AlphaFold3 output. The displayed PAE (Predicted Aligned Error) plot was generated directly using AlphaFold3 and subsequently modified solely to enhance clarity and interpretability for presentation purposes.

#### Creation of endogenous tags via CRISPR-Cas9-mediated genome editing

The *flag::dve-1* (xf398[3xflag::dve-1]), X and *lin-40::myc* (xf397[lin-40::3myc]), V strains were generated by CRISPR genome engineering. Protospacers were designed on the CRISPOR platform (http://crispor.tefor.net)^67^ and afterward confirmed using the Integrated DNA Technologies CRISPR–Cas9 guide RNA design checker. The strains were obtained by injecting the Bristol N2 strain with recombinant Cas9 protein (in house) and guide RNA molecule (IDT) as described previously.^71^ As a repair template, ssDNA oligonucleotides (IDT) were used. Each of the repair templates has 35-nucleotide-long homology arms. As a co-conversion marker, dpy-10(cn64) was used.^72^ A list of the protospacers and repair templates used is provided in supplementary data file 1.

#### RNA interference feed

RNA interference (RNAi) was performed essentially as described elsewhere.^73^ In short, the RNAi plates were supplemented with 1 mM isopropyl-β-D-thiogalactopyranoside (IPTG). HT115(DE3) *E. coli* bacteria transformed with the L4440 vector (empty vector as a negative control or carrying fragments of *dve-1* cDNA) were inoculated in LB medium overnight and seeded on IPTG nematode growth media plates. The RNAi sequences were obtained from the Ahringer RNAi library.^73^

#### Next-generation mRNA sequencing data analysis

For transcriptome analysis of *dve-1* and *lin-40* knockdown, 20 adult hermaphrodites were selected from the plates, and total RNA was extracted using TRIzol LS Reagent (Thermo Scientific) and a Monarch Spin RNA Cleanup Kit (New England Biolabs, #T2040L). RNA integrity was examined on a 2100 Bioanalyzer using an RNA 6000 Nano Kit (Agilent) and quantified using an RNA High Sensitivity Kit and a Qubit 4.0 Fluorometer (Invitrogen). NGS libraries were prepared with an Illumina TruSeq stranded mRNA LP Sample Prep Kit (Illumina, #20020594) following Illumina’s standard protocol (Part #15031047 Rev. E) but using ¼ of the reagents according to the in-house protocol of the Genomics CF (IMB Mainz). An input of 250 ng of total RNA was used, and the libraries were amplified in 10 PCR cycles. The generated libraries were profiled on a 2100 Bioanalyzer (Agilent) using a High-Sensitivity DNA Kit and quantified using a dsDNA High-Sensitivity Kit and a Qubit 4.0 Fluorometer (Invitrogen). All 16 samples were pooled together in an equimolar ratio. Sequencing was performed by the Genomics CF at IMB Mainz on an Illumina NextSeq 2000. From the pool, 750 pM with 1% PhiX was sequenced on a P2-100 flow cell, PE for 2 × 50 cycles plus 2 × 8 cycles for the dual index read.

The library quality was assessed with FastQC (version 0.11.9) and FastQScreen (version 0.15.2) before alignment against the *C. elegans* genome assembly (WS292) and its canonical gene annotations (c_elegans.PRJNA13758.WS292.canonical_geneset.gtf).^57^ Alignment was performed with STAR aligner version 2.7.3a (options: --runMode alignReads --outStd SAM --outSAMattributes NH HI AS nM MD NM --outSJfilterReads Unique --outSAMunmapped Within –outMultimapperOrder Random --outFilterMismatchNoverLmax 0.04 --outFilterMismatchNmax 999 --sjdbOverhang 74.^68^ Reads mapped to annotated features in the GTF file were counted with featureCounts version 1.6.2 using the featureCounts functionality (options: --donotsort -t exon).^69^ Finally, the overall quality of the reads and the alignment was assessed with MultiQC version 1.7.^70^

Further filtering and exploratory analysis were performed in an R framework including ggplot2.^74^ Pairwise differential expression comparisons were performed with DESeq2.^75^ Gene expression in RPKM was used to filter out individuals with a replicate average lower than 0; thus considering them as non-expressed. Differentially expressed genes (DEGs) were selected with an adjusted *p*-value (false discovery rate) of <0.01.

#### In-gel digest

In-gel digestion was performed following previously established protocols.^76^ In short, the samples were separated by electrophoresis on a 4–12% Bis-Tris gel (NuPAGE, Thermo Scientific, #NP0321) for 8 min at 180 V in 1*x* MES buffer (Thermo Scientific, #NP0002). Each lane was excised and cut into approximately 1 mm × 1 mm pieces using a sterile scalpel and then transferred into a well of a 96-well hydrophilic low protein binding filter plate (Merck Millipore, #MSBVN1210). The gel pieces were destained in destaining buffer (50% 50 mM ammonium bicarbonate buffer (ABC), pH 8.0, and 50% ethanol) at 37°C with strong agitation until complete destaining was achieved. The gel pieces were subsequently dehydrated by incubation in 100% acetonitrile for 10 min at 25°C with shaking. Reduction was carried out by incubating the gel pieces in reduction buffer (50 mM ABC, 10 mM DTT) at 56°C for 60 min. This was followed by alkylation in alkylation buffer (50 mM ABC, 50 mM iodoacetamide) for 45 min at room temperature in the dark. The gel pieces were then washed with digestion buffer (50 mM ABC) for 20 min at 25°C and dehydrated again by incubation in pure acetonitrile until the gel pieces turned white and firm. The samples were further dried at 80°C until the filter membrane of the 96-well plate appeared white. The dried gel pieces were rehydrated in a trypsin solution (50 mM ABC, 1 μg MS-grade trypsin per sample; Serva Electrophoresis #37286), and the filter plate was placed on top of a 96 deep-well collection plate (Eppendorf, #951032603). Digestion proceeded overnight at 37°C. The plate assembly was subsequently centrifuged at 300 × g for 2 min, after which the flowthrough containing the tryptic peptides was collected. This was combined with additional eluates obtained by twice treating the gel pieces with extraction buffer (50 mM ABC, 30% acetonitrile) and a further dehydration step with pure acetonitrile for 10 min at 25°C while shaking at 300 rpm, which was repeated until the gel pieces became white and hard. The peptide-containing sample was concentrated to 10% of its original volume using a Concentrator Plus (Eppendorf, #5305000304, settings V-AQ) to remove acetonitrile, followed by purification using the StageTip protocol.

#### Stage tip purification

StageTip purification was performed primarily following a previously published procedure.^77^ Desalting tips were prepared by packing two layers of C18 material (Affinisep AttractSPE, #SPE-Disk-Bio-C18-100-47.T1.20) into a 200 μL pipette tip. The tips were first primed with pure methanol. After activation, they were sequentially rinsed with solution B (80% acetonitrile, 0.1% formic acid) and solution A (0.1% formic acid) for 5 min each prior to applying the tryptic peptide samples. A final wash with solution A was then carried out. When elution was performed, the peptides were recovered using solution B. The collected samples were concentrated in a Concentrator Plus for 10 min to remove acetonitrile and then adjusted to a final volume of 14 μL with solution A.

#### MS measurement and data analysis

For MS analysis of the immunoprecipitation experiments, 5 μL of desalted and eluted peptides from each sample were injected and separated on a nanocapillary column (New Objective, 25 cm length, 75 μm inner diameter) packed in-house with C18 (Dr. Maisch GmbH) for reverse-phase chromatography. This setup was connected to an EASY-nLC 1000 system (Thermo Scientific) coupled to a Q Exactive Plus mass spectrometer (Thermo Scientific). Peptides were eluted from the column using an optimized 2-h gradient, increasing from 2% to 40% of an 80% MS-grade acetonitrile/0.1% formic acid solution at a flow rate of 225 nL/min. The mass spectrometer was operated in data-dependent acquisition mode, performing one full MS scan followed by up to ten MS/MS scans using HCD fragmentation.

For MS analysis of the proteomes from the *dve-1* RNA interference experiment, 2 μL of desalted and eluted peptides from each sample were injected and separated on an Aurora Ultimate CSI 25 × 75 C18 UHPLC column (Ionopticks) at 40°C for reverse-phase chromatography. This setup was connected to a TimsTOF HT (Bruker) interfaced with a NanoElute 2 HPLC system (Bruker). Peptides were eluted from the column using an optimized 100-min gradient, increasing from 2% to 32% of a 99.9% MS-grade acetonitrile/0.1% formic acid solution at a flow rate of 300 nL/min. The mass spectrometer was operated in data-dependent acquisition PASEF standard mode.

Protein analysis was performed as previously described.^35^ In brief, contaminants, reverse database hits, protein groups identified only by site, and protein groups with fewer than two peptides (with at least one classified as unique) were filtered out from the MaxQuant proteinGroups.txt file. Missing values were imputed by shifting a beta distribution, based on the LFQ intensity values, to the limit of quantitation. Volcano plots were generated from RStudio using ggplot2 and other packages. The threshold for protein enrichment was set at a fold change > |4| and a *p*-value <0.05 (Welch’s *t* test), unless otherwise specified.

#### Functional enrichment analysis

Genes were queried in the Gene Ontology database using the ClusterProfiler R package.^52^^,^^53^ Terms found among the enriched genes or proteins were tested for over-representation with an adjusted *p*-value (false discovery rate) < 0.05 (Fisher’s exact test) against terms found in the background (defined as all quantified genes or proteins in the comparison, whether enriched or not).

#### Real-time quantitative PCR (RT‒qPCR)

RNA from gravid adults was extracted as described in the “Next-generation mRNA sequencing data analysis” section. Extracted RNA was subjected to DNase I (Zymo Research, #E1010). Two sets for cDNA synthesis were prepared, each with 500 ng of RNA and SuperScript IV Reverse Transcriptase (Invitrogen, #18090010). The first reverse transcription reaction contained oligo d(T) primers for the synthesis of cDNA from the housekeeping gene *tba-1*. A second reaction contained primers with repetitive telomeric sequences to ensure TERRA amplification. All primer sequences are provided in supplementary data file 1.

RT‒qPCR was carried out using Power SYBR Green PCR Master Mix (Invitrogen, #4368706) and the QuantStudio 5 System (Applied Biosystems). Published primer sequences for *tba-1* and TERRA were used for qPCR.^55^ For each sample, the delta between the Cq value of the target gene and the Cq value of the housekeeping gene was calculated (ΔCq). The ΔΔCq values were calculated by normalization to the ΔCq mean of the L4440 empty vector negative control, and 2^−ΔΔCT^ was plotted as relative quantification (RQ) values.

#### Assay for transposase-accessible chromatin using sequencing (ATAC-Seq)

RNA from wild-type *C. elegans* (N2 (Bristol)) was grown at 20°C in the indicated RNAi strain as described previously.^73^^,^^78^ Synchronized L1 larvae were obtained by bleaching gravid adults and leaving the recovered eggs to hatch overnight at room temperature in M9 medium. Synchronized L1s were transferred onto NGM plates supplemented with 4 mM IPTG and 100 μg/mL carbenicillin and seeded with either the control (L4440 vector) or RNAi against *dve-1* and grown until the gravid adult stage.

Embryos were isolated from RNAi-treated gravid adults by bleaching. For each biological replicate, 3 × 8000 embryos/1.5 mL tube were collected and resuspended in embryo buffer (118 mM NaCl, 25 mM HEPES (pH 7.3), 48 mM KCl, 2 mM CaCl_2_, and 2 mM MgCl_2_). Eggshells were digested with chitinase (2 U/mL; Sigma Aldrich) for 10–15 min with rotation at room temperature, followed by mechanical disruption by pipetting. The reaction was quenched by the addition of L-15 medium (Life Technologies) supplemented with 10% fetal bovine serum (PAN Biotech). The cell suspensions were centrifuged briefly (100 × g for 1 min at 4°C) to remove eggshell debris, and the supernatant containing the released cells was pelleted at 1000 × g for 10 min at 4°C. Cells were resuspended in L-15 medium without serum and filtered through a 30 μm cell strainer. Live cells were sorted based on forward and side scatter with DAPI (Sigma Aldrich) exclusion using a BD FACSAria III SORP (P#210144599) cell sorter.

ATAC-Seq was performed on 100000 sorted cells following the Omni-ATAC protocol (PMID: 28846090). The sorted cells were centrifuged at 100 × g for 10 min on a 4°C swinging-bucket rotor and then were washed three times with ATAC-Resuspension Buffer (RSB) (10 mM Tris-HCl (pH 7.4), 10 mM NaCl, and 3 mM MgCl_2_) containing 0.1% NP40, 0.1% Tween 20, and 0.01% Digitonin. The resulting nuclei were pelleted at 1000 × g for 10 min at 4°C, after which the supernatant was carefully removed. The pelleted nuclei were then resuspended in 50 μL of transposition mix, which contained 25 μL of 2× TD buffer (Tris-HCl (pH 7.6), 10 mM MgCl_2_, 20% dimethylformamide, and water), 1.67 μL of in-house-prepared Tn5 transposase (IMB Protein Production) (100 nM final), 16.5 μL of PBS, 0.25 μL of 2% digitonin, 0.5 μL of 10% Tween 20 and 6.08 μL of water. The transposition reactions were incubated for 30 min at 37°C with shaking at 600 rpm. DNA fragments were purified using a Zymo DNA Clean & Concentrator Kit, and the remainder of the ATAC-Seq library preparation was performed as originally described.^79^ Sequencing was performed using the Illumina NextSeq 2000 platform with 100 cycles and paired-end reads. Reads were trimmed for adapter sequences using TrimGalore version 0.6.10 and aligned with Bowtie2 version 2.4.5 on ce10.

#### Western blotting

Protein samples were denatured in 1*x* LDS at 70°C for 10 min and subsequently loaded on 4–12% Bis-Tris gradient gels (NuPAGE, Thermo Scientific). Electrophoresis was carried out at a constant voltage of 180 V for 30 min in 1*x* MES buffer. Following separation, the gels were briefly rinsed with water and equilibrated in transfer buffer (25 mM Tris, 192 mM glycine, 20% ethanol). Nitrocellulose membranes (Amersham Protran, VWR) were also equilibrated in transfer buffer prior to assembly. The transfer stack was prepared by layering the gel and membrane between pre-moistened Whatman paper and submerged in a Bio-Rad blotting tank with ice-cold transfer buffer supplemented with an external cooling block. The proteins were transferred onto the membrane at 300 mA for 60 min. For immunodetection, the membranes were blocked with PentaHis Blocking Solution (Qiagen, #34460) for 1 h at room temperature. After three 5-min washes in TBS-T buffer (1*x* TBS, 0.1% Tween 20, 0.5% Triton X-100), the membranes were incubated for 1 h with anti-His-HRP-conjugated antibody (Qiagen, #34460) diluted 1:1000 in blocking solution. Post-incubation, the membranes were washed three times with TBS-T and treated with a chemiluminescent substrate (Thermo Scientific SuperSignal West Pico PLUS Chemiluminescent Substrate, #15626144) for signal development. Chemiluminescent detection was performed using a ChemiDoc XRS+ imaging system (Bio-Rad), and images were acquired and processed with Image Lab 6.1.0 software.

#### Brood size and viability

For brood size determination, N2 embryos were isolated from *dve-1* or L4440 empty vector-expressing HT115 *E. coli* lawn cultures grown at 20°C. After reaching adulthood, the worms were transferred to a new plate every day until no eggs were laid for two consecutive days. Viable progeny were counted approximately 24 h after the parent was removed. P-values were calculated by two-sided Welch’s *t* test (∗∗*p*-value <0.005; ∗∗∗∗*p*-value <0.0001) (*n* = 4).

#### Microscopy

For fluorescence microscopy of DVE-1::GFP and POT-1::mCherry whole worms, approximately 10 μL of M9 buffer was dispensed onto a microscope slide. Ten worms were transferred first to unseeded plates and then to the droplet, minimizing the carryover of the bacterial lawn. Worms were fixed by the addition of 15 μL of acetone applied directly to the M9 droplet. Once the acetone evaporated completely, fixation was repeated with a second application of acetone. Following evaporation of the second acetone addition, worms were immediately rehydrated using 10 μL of PBS-T (0.1% Triton X-100) and incubated for at least 10 min to allow sufficient rehydration. Approximately 8 μL of DAPI-containing mounting medium (Vector Laboratories, #H-1200-10) was subsequently added, and a coverslip was applied. The samples were incubated at room temperature for 15 min before being sealed with nail polish to prevent drying. The slides were incubated overnight at 4°C and imaged the next day. Animals were imaged using a TCS SP5 Leica confocal microscope or a Leica STELLARIS 8 FALCON equipped with an HCX PL APO 63*x* oil objective, Leica hybrid detectors (HyD), and the acquisition software Leica LASX AF.

#### Evolutionary analysis of DVE-1 across nematodes

To determine whether the telomere-binding domain of DVE-1 is conserved beyond *C. elegans*, we applied a multi-layered comparative evolutionary framework integrating orthology inference, gene phylogeny, domain architecture conservation and a bit-motif-score analysis. The reference DVE-1 protein from *C. elegans* (UniProt Q86MI0) was retrieved from UniProt and mapped to its corresponding Ensembl Metazoa gene identifier. Orthology relationships were obtained from the Ensembl Metazoa database (release 62). In parallel, complete proteomes were downloaded from Ensembl Metazoa BioMart for *Ascaris suum*, *Brugia malayi*, *Caenorhabditis brenneri*, *C. briggsae*, *C. elegans*, *C. japonica*, *C. remanei*, *Loa loa*, *Necator americanus*, *Onchocerca volvulus*, *Pristionchus pacificus*, *Strongyloides ratti*, *Trichinella spiralis*, and *Trichuris muris*. To further validate vertical inheritance, we compared the DVE-1 gene phylogeny with that of the established nematode species tree. The gene trees were consistent with the species phylogeny, suggesting descent from a single ancestral copy and consistent with functional conservation.^43^ To assess whether the domain architecture is conserved across nematodes, Pfam and SMART domain coordinates were extracted for each ortholog and merged into a non-redundant, ordered domain architecture by resolving overlapping annotations. The sequences used for the conservation comparison of residues within the second homeobox domain of DVE-1 corresponded to the same set of DVE-1 homologs employed in the Comparative Domain Architecture Analysis of DVE-1 and in the Gene Phylogeny and Concordance with Species Relationships analyses. From these sequences, the region corresponding to the second homeobox domain was extracted and aligned. The resulting alignment was subsequently processed using Observed Counts in Skylign^80^ to generate a bit score motif analysis. The parameters used in Skylign were logos generated from the full alignment, letter heights determined by score, and alignment processing based on observed counts.

### Quantification and statistical analysis

Details about quantification and statistical analysis can be found in the respective method details “Next-generation mRNA sequencing data analysis”, “MS measurement and data analysis”, “Functional enrichment analysis”, “Real-time quantative PCR (RT-PCR)”, “Brood size and viability” and “Assay for Transposase-Accessible Chromatin using Sequencing (ATAC-Seq)”.

In short, the Next-generation mRNA sequencing data analysis was performed in quadruplicates and pairwise differential expression comparisons were performed. Gene expression in RPKM was used to filter out individuals with a replicate average lower than 0; thus considering them as non-expressed. Differentially expressed genes (DEGs) were selected with an adjusted *p*-value (false discovery rate) of <0.01.

MS measurement and data analysis was carried out in quadruplicates. Contaminants, reverse database hits, protein groups identified only by site, and protein groups with fewer than two peptides (with at least one classified as unique) were filtered out from the MaxQuant proteinGroups.txt file. Missing values were imputed by shifting a beta distribution, based on the LFQ intensity values, to the limit of quantitation. Volcano plots were generated from RStudio using ggplot2 and other packages. The threshold for protein enrichment was set at a fold change > |4| and a *p*-value <0.05 (Welch’s *t* test), unless otherwise specified.

For functional enrichment analysis, terms found among the enriched genes or proteins were tested for over-representation with an adjusted *p*-value (false discovery rate) < 0.05 (Fisher’s exact test) against terms found in the background.

Real-time quantative PCR (RT-PCR) was performed in quadruplicates. P-values were calculated by two-tailed Welch’s *t* test (∗*p*-value <0.05).

Brood size and viability was carried out in quadruplicates. P-values were calculated by two-sided Welch’s *t* test (∗∗*p*-value <0.005; ∗∗∗∗*p*-value <0.0001).

ATAC-Seq was carried out in triplicates. DNA sequences with varying numbers of telomere sequence repeats were mapped to the total reads and counted. Based on this, the mean of each DNA sequence was calculated for the respective target proteins.

## Supplemental information

Document S1. Figures S1–S5

Table S1. Oligonucleotides

Table S2. DVE1 IP enriched proteinGroup

Table S3. DVE1 IP reduced proteinGroups

Table S4. LIN40 IP enriched proteinGroups

Table S5. LIN40 IP reduced proteinGroups

Table S6. TELO PD enriched proteinGroups

Table S7. TELO PD reduced proteinGroups

## Footnotes

Supplemental information can be found online at https://doi.org/10.1016/j.isci.2026.117120.
