# Supplementary material for: DVE-1 is a telomere-binding protein and links the NuRD complex to telomere regulation in C. elegans
Source: iScience. 2026 Aug 24;29(9):117120. doi: 10.1016/j.isci.2026.117120 (PMC13524731; doi:10.1016/j.isci.2026.117120)
Supplement: Supplementary file 1 [file mmc1.pdf]

iScience, Volume *29*

Supplemental information

DVE-1 is a telomere-binding protein and links the NuRD complex to telomere regulation in *C. elegans*

Jan Sluka, Alexandra Blake, Nadezda Podvalnaya, Albert Fradera-Sola, Sabrina Dietz, Lars Teschke, Alejandro Ceron-Noriega, Rosa Herrera-Rodriguez, Valerie Arz, René Ketting, Jan Padeken, Emily Nischwitz, and Falk Butter

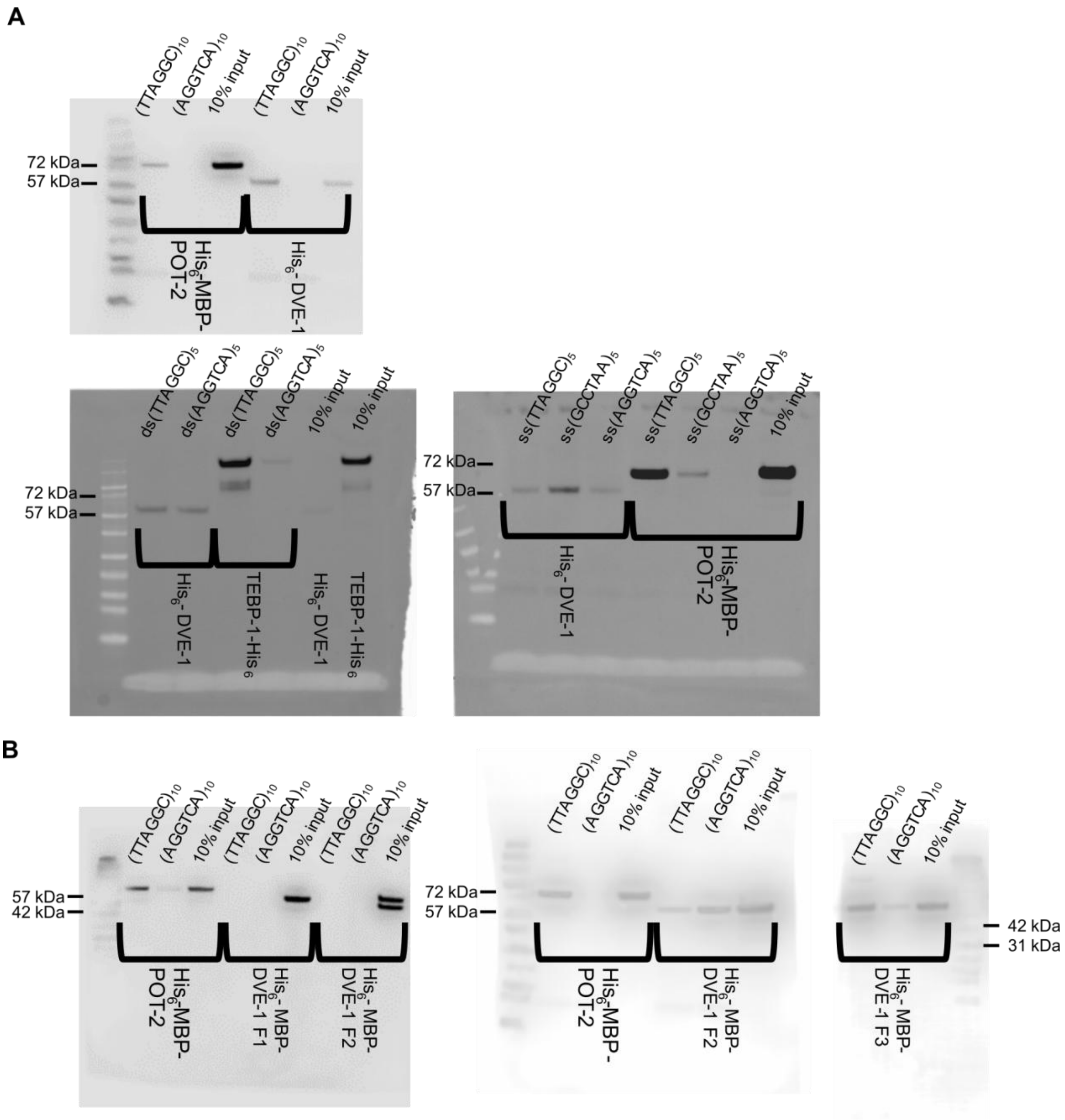

Figure S1. (**A**) Uncropped western blots from Figure 1 of DNA pulldowns with His_6-tagged DVE-1 from *E. coli* lysates using concatenated, single-stranded or double-stranded biotinylated DNA oligonucleotide baits with telomeric (TTAGGC) and (GCCTAA) or control (AGGTCA) sequences. Recombinantly expressed POT-2 was used as a known single-strand telomeric binder, and TEBP-1 was used as a known double-strand binder. MBP: maltose-binding protein. (**B**) Uncropped western blots from Figure 1 of a DNA pull- down experiment as described in (A) with the recombinantly expressed DVE-1 fragments and POT-2 as a positive control. The pull-down for Fragment 2 was repeated due to the unexpected appearance of a second band. For Figure 1 of DVE-1 fragment 2, the image of the western blot in the center was used.

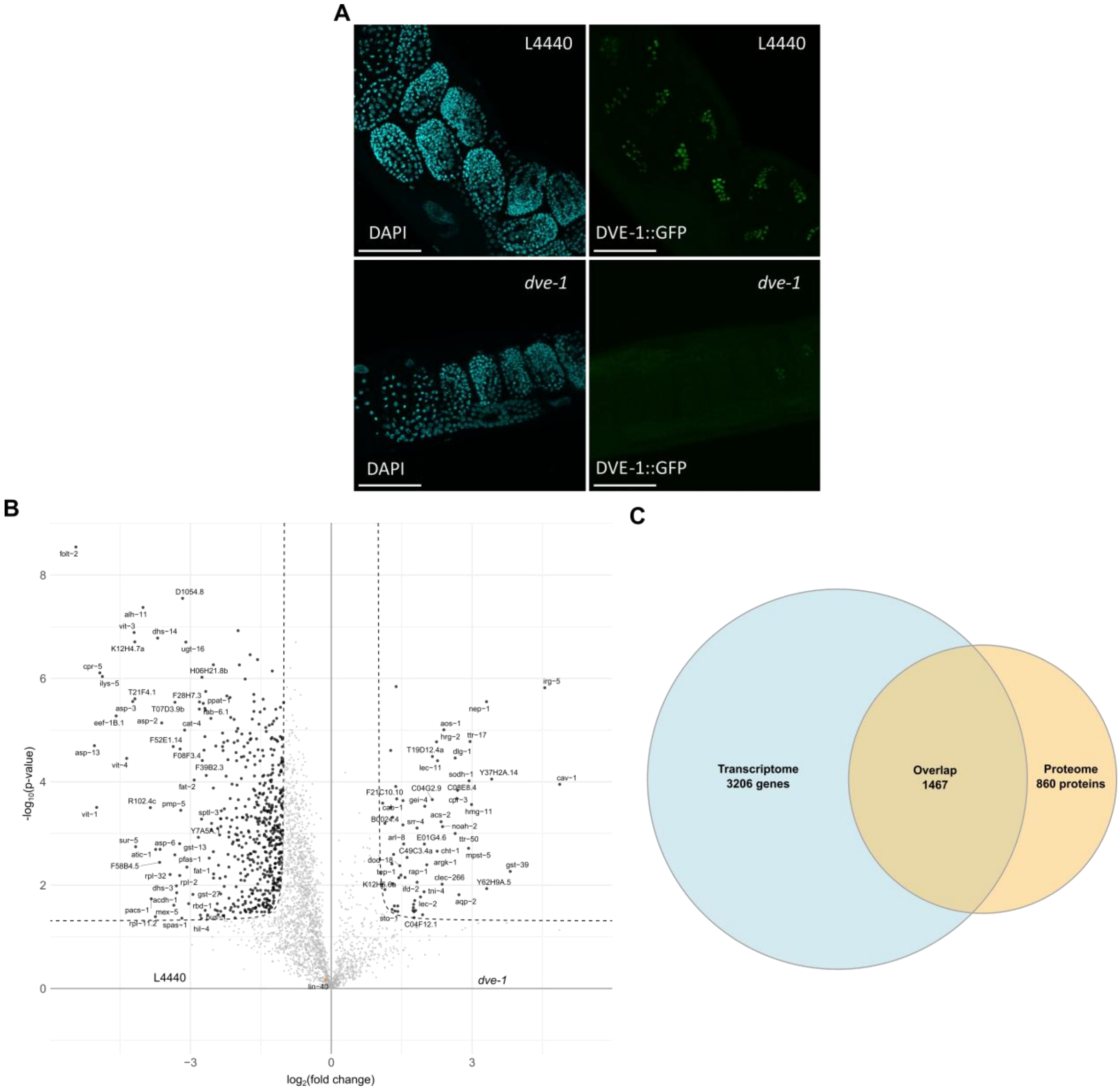

Figure S2. (**A**) Maximum intensity projections of representative confocal z-stacks of embryos in the germline expressing transgenic DVE-1::GFP. Scale bars are 50 µm. (**B**) Volcano plot of the *dve-1* RNAi knockdown proteome against the L4440 empty vector control from gravid adult extracts of *C. elegans*. Proteome measurements were performed in quadruplicates for each condition. Enriched proteins (enrichment threshold > 2-fold, p-value < 0.05) are labeled as black dots. Proteins below the threshold are depicted as gray dots. (**C**) Venn diagram of the overlapping downregulated genes in the transcriptome (blue) and proteome (yellow) of the *dve-1* knockdown.

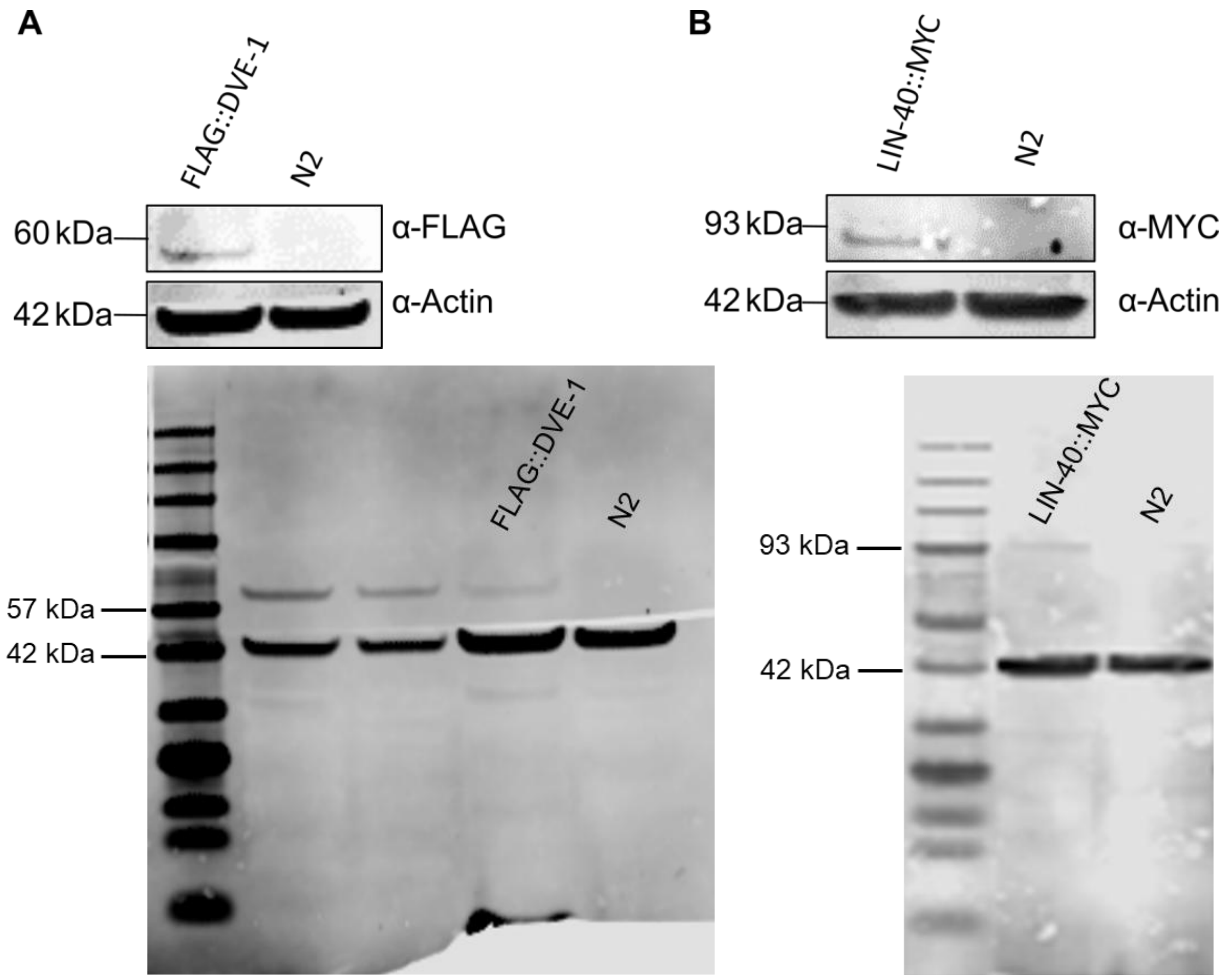

Figure S3. (**A**) Western blot analysis of endogenous FLAG-tagged DVE-1 (60 kDa) with the wild-type N2 strain used as a negative control and an actin reference protein. The top image shows the cropped western blot, and the bottom image shows the uncropped western blot with a marker. (**B**) Western blot analysis of endogenous MYC-tagged LIN-40 (93 kDa), with the wild-type N2 strain used as a negative control and an actin reference protein. The top image shows the cropped western blot, and the bottom image shows the uncropped western blot with a marker.

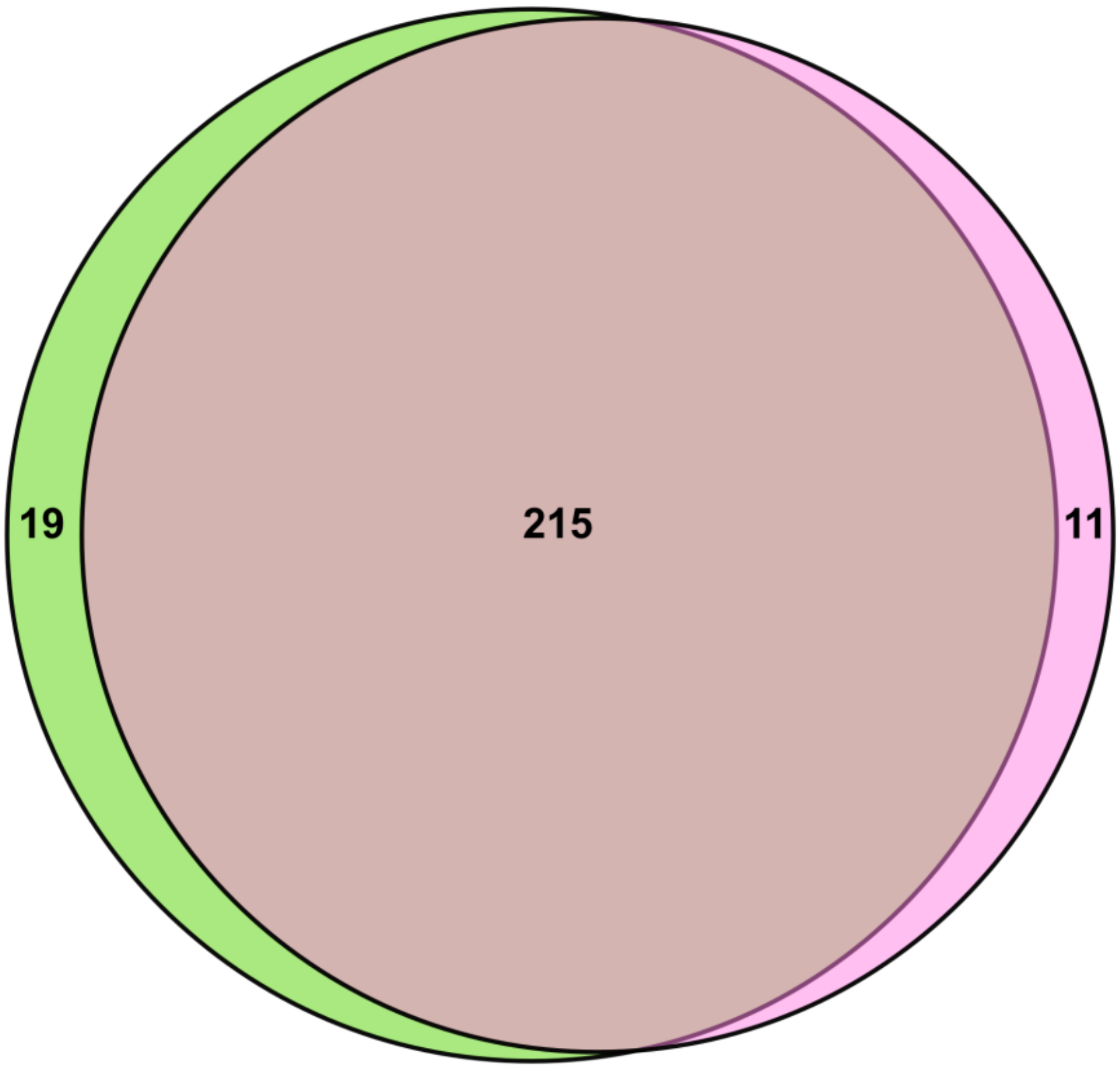

Figure S4. Venn diagram illustrating foci overlap between POT-1::mCherry (purple) and DVE-1::GFP (green). Among 234 DVE-1::GFP foci, 215 (95%) co-localized with 226 POT-1::mCherry foci. Foci were quantified across three independent embryo images.

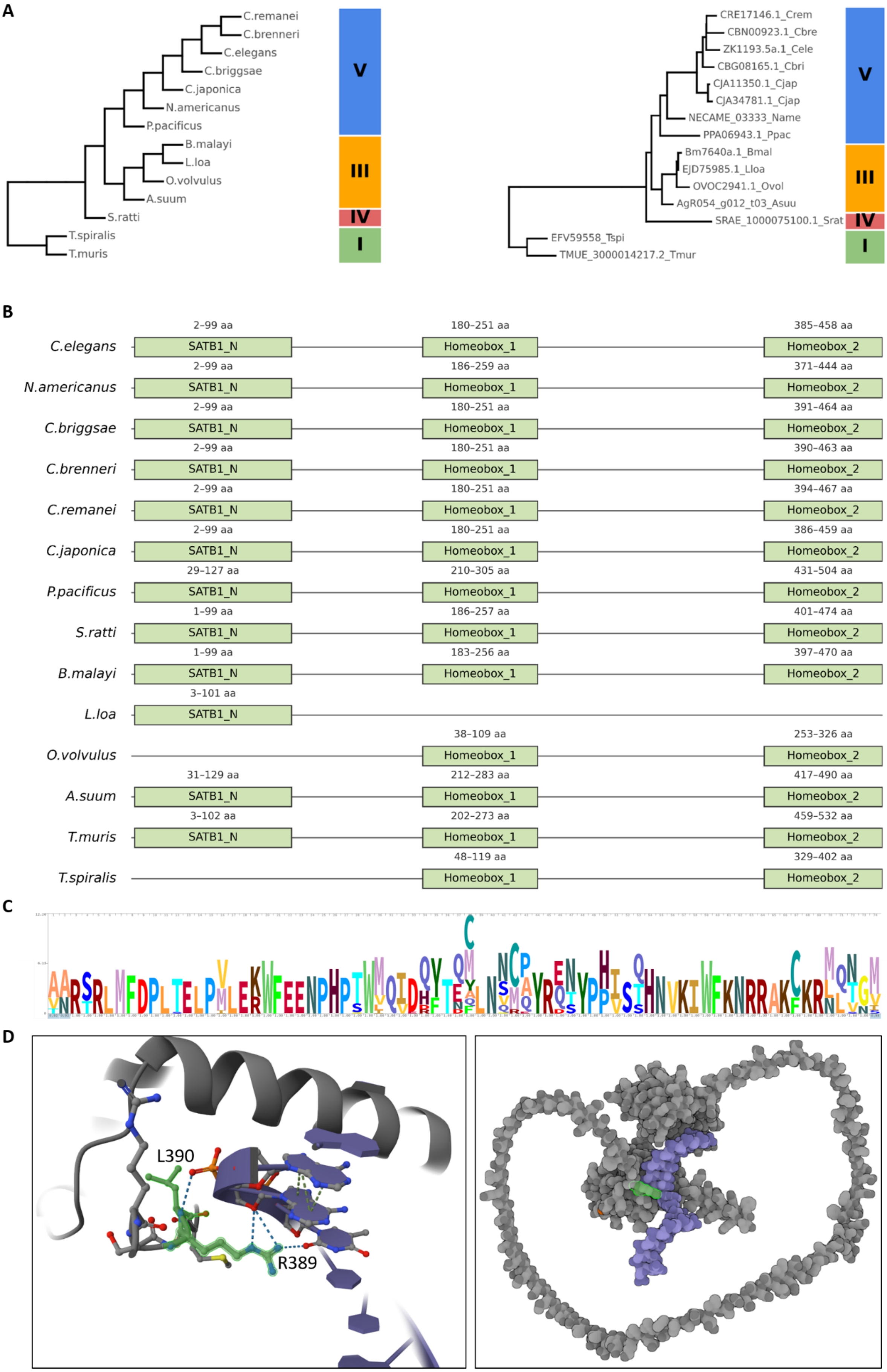
A
C.remanei
C.brenneri
C.elegans
C.briggsae
C.japonica
N.americanus
P.pacificus
B.malayi
L.loa
O.volvulus
A.suum
S.ratti
T.spiralis
T.muris
V
III
IV
I
CRE17146.1_Crem
CBN00923.1_Cbre
ZK1193.5a.1_Cele
CBG08165.1_Cbri
CJA11350.1_Cjap
CJA34781.1_Cjap
NECAME_03333_Name
PPA06943.1_Ppac
Bm7640a.1_Bmal
EJD75985.1_Lloa
OVOC2941.1_Ovol
AgR054_g012_t03_Asuu
SRAE_1000075100.1_Srat
EFV59558_Tspi
TMUE_3000014217.2_Tmur
V
III
IV
I
B
C.elegans: SATB1_N 2–99 aa; Homeobox_1 180–251 aa; Homeobox_2 385–458 aa
N.americanus: SATB1_N 2–99 aa; Homeobox_1 186–259 aa; Homeobox_2 371–444 aa
C.briggsae: SATB1_N 2–99 aa; Homeobox_1 180–251 aa; Homeobox_2 391–464 aa
C.brenneri: SATB1_N 2–99 aa; Homeobox_1 180–251 aa; Homeobox_2 390–463 aa
C.remanei: SATB1_N 2–99 aa; Homeobox_1 180–251 aa; Homeobox_2 394–467 aa
C.japonica: SATB1_N 2–99 aa; Homeobox_1 180–251 aa; Homeobox_2 386–459 aa
P.pacificus: SATB1_N 29–127 aa; Homeobox_1 210–305 aa; Homeobox_2 431–504 aa
S.ratti: SATB1_N 1–99 aa; Homeobox_1 186–257 aa; Homeobox_2 401–474 aa
B.malayi: SATB1_N 1–99 aa; Homeobox_1 183–256 aa; Homeobox_2 397–470 aa
L.loa: SATB1_N 3–101 aa
O.volvulus: Homeobox_1 38–109 aa; Homeobox_2 253–326 aa
A.suum: SATB1_N 31–129 aa; Homeobox_1 212–283 aa; Homeobox_2 417–490 aa
T.muris: SATB1_N 3–102 aa; Homeobox_1 202–273 aa; Homeobox_2 459–532 aa
T.spiralis: Homeobox_1 48–119 aa; Homeobox_2 329–402 aa
C
D
L390
R389

Figure S5. (**A**) Concordance between nematode species and DVE-1 gene phylogenies. The left tree shows a species tree for the 12 nematode species showing orthologousrelationships with DVE-1, representing four of the five recognized nematode clades, with no orthologs detected in clade II. Clade membership is indicated by stacked rectangles at the tips: clade V (blue; *Caenorhabditis remanei*, *C. brenneri*, *C. elegans*, *C. briggsae*, *C. japonica*, *Necator americanus*, and *Pristionchus pacificus*), clade III (orange; *Brugia malayi*, *Loa loa*, *Onchocerca volvulus*, and *Ascaris suum*), clade IV (red; *Strongyloides ratti*), and clade I (green; *Trichinella spiralis* and *Trichuris muris*). The right panel presents the gene tree of DVE-1 orthologs from the same taxa, mirroring the species-level topology; clade membership is shown using the same color-coded rectangles as in the species tree. (**B**) Conserved DVE-1 domain architecture across nematodes. Schematic showing the domain organization of DVE-1 orthologs across nematode species, extending Figure 1C. DVE-1 proteins contain an N-terminal SATB1N domain followed by two homeobox domains (Homeobox 1 and Homeobox 2), with Homeobox 2 proposed to mediate telomere binding. Domains are vertically aligned by name across species to facilitate comparison, but the schematic is not drawn to scale. Amino acid coordinates of each domain within the protein are indicated above the boxes. The figure reports domain presence or absence only and is used to evaluate the conservation of the DVE-1 domain architecture among one-to-one orthologs across nematodes. (**C**) Bit-score-motif of the second homeobox of DVE-1 among the species listed in (B), starting from aa385. (**D**) Structural prediction of DVE-1 (gray) with C-rich telomeric DNA repeats (purple) with AlphaFold3. The left panel shows the interaction of the amino acids arginine 389 and leucine 390 (highlighted in green) with telomeric DNA at the molecular level. The right panel illustrates the full protein structure (gray) in relation to the telomeric DNA (purple). The amino acids arginine 389 and leucine 390 are highlighted in green. The images are based on the same model prediction as the AlphaFold3 analysis from Figure 1B.
